# Unprecedented enantio-selective live-cell mitochondrial DNA super-resolution imaging and photo-sensitizing by the chiral ruthenium polypyridyl DNA “light-switch”

**DOI:** 10.1093/nar/gkad799

**Published:** 2023-11-02

**Authors:** Rong Huang, Chun-Hua Huang, Jing Chen, Zhu-Ying Yan, Miao Tang, Jie Shao, Kaiyong Cai, Ben-Zhan Zhu

**Affiliations:** Key Laboratory of Biorheological Science and Technology, Ministry of Education, College of Bioengineering, Chongqing University, Chongqing 400044, China; State Key Laboratory of Environmental Chemistry and Ecotoxicology, Research Center for Eco-Environmental Sciences, and University of Chinese Academy of Sciences, Chinese Academy of Sciences, Beijing 100085, China; State Key Laboratory of Environmental Chemistry and Ecotoxicology, Research Center for Eco-Environmental Sciences, and University of Chinese Academy of Sciences, Chinese Academy of Sciences, Beijing 100085, China; State Key Laboratory of Environmental Chemistry and Ecotoxicology, Research Center for Eco-Environmental Sciences, and University of Chinese Academy of Sciences, Chinese Academy of Sciences, Beijing 100085, China; State Key Laboratory of Environmental Chemistry and Ecotoxicology, Research Center for Eco-Environmental Sciences, and University of Chinese Academy of Sciences, Chinese Academy of Sciences, Beijing 100085, China; State Key Laboratory of Food Science and Resources, Jiangnan University, Wuxi 214122, China; State Key Laboratory of Environmental Chemistry and Ecotoxicology, Research Center for Eco-Environmental Sciences, and University of Chinese Academy of Sciences, Chinese Academy of Sciences, Beijing 100085, China; State Key Laboratory of Environmental Chemistry and Ecotoxicology, Research Center for Eco-Environmental Sciences, and University of Chinese Academy of Sciences, Chinese Academy of Sciences, Beijing 100085, China; Key Laboratory of Biorheological Science and Technology, Ministry of Education, College of Bioengineering, Chongqing University, Chongqing 400044, China; State Key Laboratory of Environmental Chemistry and Ecotoxicology, Research Center for Eco-Environmental Sciences, and University of Chinese Academy of Sciences, Chinese Academy of Sciences, Beijing 100085, China; College of Environmental Science and Technology, Shandong University, Qingdao, Shandong 266237, PR China

## Abstract

Mitochondrial DNA (mtDNA) is known to play a critical role in cellular functions. However, the fluorescent probe enantio-selectively targeting live-cell mtDNA is rare. We recently found that the well-known DNA ‘light-switch’ [Ru(phen)_2_dppz]Cl_2_ can image nuclear DNA in live-cells with chlorophenolic counter-anions via forming lipophilic ion-pairing complex. Interestingly, after washing with fresh-medium, [Ru(phen)_2_dppz]Cl_2_ was found to re-localize from nucleus to mitochondria via ABC transporter proteins. Intriguingly, the two enantiomers of [Ru(phen)_2_dppz]Cl_2_ were found to bind enantio-selectively with mtDNA in live-cells not only by super-resolution optical microscopy techniques (SIM, STED), but also by biochemical methods (mitochondrial membrane staining with Tomo20-dronpa). Using [Ru(phen)_2_dppz]Cl_2_ as the new mtDNA probe, we further found that each mitochondrion containing 1–8 mtDNA molecules are distributed throughout the entire mitochondrial matrix, and there are more nucleoids near nucleus. More interestingly, we found enantio-selective apoptotic cell death was induced by the two enantiomers by prolonged visible light irradiation, and in-situ self-monitoring apoptosis process can be achieved by using the unique ‘photo-triggered nuclear translocation’ property of the Ru complex. This is the first report on enantio-selective targeting and super-resolution imaging of live-cell mtDNA by a chiral Ru complex via formation and dissociation of ion-pairing complex with suitable counter-anions.

## Graphical Abstract

**Graphical Abstract:**
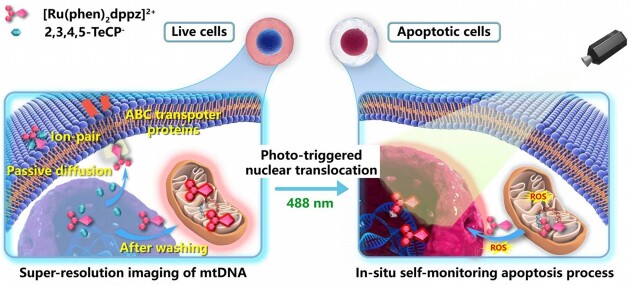

## Introduction

Since DNA was discovered as the genetic carrier, research towards the illustration of the cellular DNA structure has become very important. The copy number and functional changes of mitochondrial DNA (mtDNA) can directly affect energy conversion of the body's internal systems, brain function, metabolic regulation, cellular canceration, immune activation, aging rate and life span. Therefore, changes in mtDNA content and function can be used as biomarkers for many diseases (1,2). Fluorescence microscopy is the key bio-imaging tool applied to observe cellular DNA structure (3–5). However, because of the light diffraction limit, the spatial resolution of optical microscopy is lower than 250 nm, which cannot afford the demand of sub-cellular scale observations. In recent years, the advancement of super resolution microscopy (SRM) has broken this technical bottleneck (3,6,7). Stimulated emission depletion (STED) microscopy and structured illumination microscopy (SIM) are two of the widely applied methods to SRM, which provide contrasting features (8,9). SIM is based on the illumination of sample with patterned light, and then the information in the Moire fringes that lie outside of the normal range of observation is analyzed. It provides a resolution limit of about 100 nm. SIM combines imaging speed with improved resolution, meaning that this technique is the best choice for live cell imaging, 3D imaging and thick tissue sections (9,10). By selective quenching photo-excited probe by a depletion beam, STED gets higher resolution about 30 nm (11). But require probes with stringent photo-stability and/or photo-excitation properties.

Most of commercial nucleus and mtDNA dyes are organic dyes, which suffer from small Stokes shifts, photo-bleaching and short emission lifetimes, thus they cannot afford the demand of SRM imaging. In particular, the probe for live cell mtDNA is rare. Commercially available mitochondria dyes such as MitoTracker probes and JC-1 are not specifically targeting mtDNA. So, it is highly needed to develop novel fluorescent probes for SRM mtDNA staining. Transition metal complexes such as ruthenium, osmium and iridium polypyridyl imaging agents for disease diagnosis and cellular organisms have many potential advantages over conventional probes, such as long emission lifetimes, large Stokes shifts and good photo-stability (12–20). They can also be applied as photosensitizers, photo-catalyst and luminescent theranostic toolkits (21–24). During our studies on synergism of biochemical and toxicological interactions between inorganic and organic compounds, (25–39). We recently found that [Ru(phen)_2_dppz]Cl_2_ was suitable for imaging the structure of cellular nuclear DNA in the presence of chlorophenols by confocal laser scanning microscopy (CLSM) (40–42). We further found that the nuclear uptake of Ru(II)-polypyridyl cationic complexes facilitated by chlorophenolate counter-anions was correlated positively with the binding stability but inversely with the lipophilicity of the formed ion pairs (40,43), and the efflux of nuclear [Ru(phen)_2_(dppz)]^2+^ was mediated mainly via ATP-Binding Cassette transporter proteins (44). Moreover, their enantiomers can image nucleus even nucleolus by fluorescence lifetime imaging microscopy (FLIM) (45,46). Ru(II)-polypyridyl complexes should also be ideal dyes for transmission electron microscopy (TEM) imaging owing to their high electron-dense ruthenium center similar with its close Os(II) analogs. Os(II) polypyridyl complexes also function as an unparalleled enantioselective nuclear DNA imaging reagent especially suitable for correlative light and electron microscopy (CLEM) studies in both living and fixed cells (47,48). In particular, recent studies demonstrated that ruthenium complexes can also be applied as cellular STED probes (49,50). Ruthenium and iridium complexes have also been investigated as mitochondria imaging agents and mitochondria targeting anticancer drugs (51,52). A Ru(II) peptide conjugate was reported to image and photo-induce damage of mitochondrial DNA (53). However, they did not show direct evidence that it can bind to mtDNA in cells, they just co-localized it with mito-tracker stain and imaged by fluorescence microscopy. As we know, one additional major shortcoming for Ru peptide conjugate is that it suffers laborious synthetic process, even if it works. A dinuclear Ru complex was reported to super-resolution image of nuclear chromatin and mitochondria, but it stained mitochondrial membrane but not mtDNA (50). It is known that Ir complexes have luminescence by themselves, with only small fluorescence increase after binding with DNA. Besides, they usually show shorter excitation wavelength than Ru complexes. Most of previous reports on mitochondrial targeting Ir(III) complexes have focused on the anticancer ability but not their super-resolution mtDNA imaging ability (54,55). In short, the exact location of Ru complexes in mitochondria (mtDNA, intermembrane space or proteins) remains unclear.

Herein, we investigated the application of ruthenium polypyridyl complexes for SIM and STED technique, especially the application of [Ru(phen)_2_dppz]Cl_2_ for the first time as live cell mtDNA SR probe. Through formation and dissociation of ion-pair complex with chlorophenolic counter-anions, we examined the capacity of [Ru(phen)_2_dppz]Cl_2_ for both directing the complex to cellular organelles (nucleus and mitochondria) and their suitability as SIM and STED probes. We confirmed the mtDNA staining ability of the Ru complex not only by super-resolution optical microscopy techniques such as SIM and STED in live cells, but also by biochemical methods such as agarose gel electrophoresis and mitochondrial membrane stained with Tomo20-dronpa. What is more, competitive binding with the classical mtDNA intercalating dye PicoGreen indicated that [Ru(phen)_2_dppz]Cl_2_ intercalated into mtDNA. We found that [Ru(phen)_2_dppz]Cl_2_ produces SIM and STED images of mtDNA and nuclear DNA at high resolution because of a unique combination of its extreme photo-stability and DNA binding selectivity. The chirality of the Ru(II) polypyridyl complex not only influence its cellular DNA luminescent intensity, but also determine its cellular phototoxicity efficiency. The Δ-enantiomer of Ru(II) complex displayed much higher luminescence intensity in nucleus and mitochondria than the Λ-enantiomer. Further we found that the Δ-enantiomer also showed stronger phototoxic effects to cells than the Λ-enantiomer.

## Materials and methods

### Chemicals

The [Ru(phen)_2_(dppz)]Cl_2_ complex was synthesized according to references (56–58), chlorophenols were purchased from Sigma. MitoTracker Green and To-pro-3 probes were purchased from Life technologies.

### Separation of the Two Enantiomers

The enantiomers of [Ru(phen)_2_(dppz)]Cl_2_ were separated using a CYCLOBOND I 2000 DMP high-performance liquid chromatography (HPLC) column (250 × 21.2 mm, 5 μm, Sigma) on a Agilent 1260 HPLC. Samples were eluted at a flow rate of 20 ml/min with an isocratic solvent composition of 36/12/52 (v/v/v) 30 mM NH_4_PF_6_ (aq.):CH_3_CN:EtOH. The fractions were monitored at 268 nm. The Δ-enantiomer eluted first, followed by the Λ-isomer. The fractions of the two enantiomers were collected manually with heart cutting. Then, the collected solution was evaporated under reduced pressure and re-dissolved in acetone to a saturated solution. The chloride salt of the compound was precipitated from the saturated solution by the addition of an acetone solution of tetra-n-butylammonium chloride (1 M). The solids were filtered off, washed with acetone and diethyl ether, dried (59). Assignment of the two fractions was confirmed by circular dichroism. The optical purity of enantiomers was estimated as 98% ee for Δ-enantiomer and 98% ee for Λ-enantiomer by HPLC analysis (250 × 4.6 mm, 5 μm, CYCLOBOND I 2000 DMP, Sigma).

### Cell culture

Human cervical cancer (HeLa) cells were cultured in DMEM medium with 10% fetal bovine serum and 1% penicillin-streptomycin, at 37°C under a 5% CO_2_ atmosphere. Cells for confocal imaging were seeded on round coverslips at a density of ∼100 000 cells/coverslip and cultured for 1 day.

### Cellular treatments with Ru complex or 2,3,4,5-TeCP

#### Nuclear imaging

Cells incubated with 100 μM [Ru(phen)_2_(dppz)]Cl_2_ and 300 μM 2,3,4,5-TeCP for 0.5 h, washed with PBS for 3 times and incubated with fresh medium for 0 h.

#### Mitochondria imaging

Cells incubated with 100 μM [Ru(phen)_2_(dppz)]Cl_2_ and 300 μM 2,3,4,5-TeCP for 0.5 h, washed with PBS for 3 times and incubated with fresh medium for 3 h.

### Confocal laser scanning microscopy (CLSM)/ Structure illumination microscopy (SIM)

After incubated with Ruthenium complexes, cells were rinsed with PBS for 3 times. Different treatment of cells was luminescent imaged on a CLSM using 40 × oil-immersion lens for slide imaging, or on a super-resolution SIM using 100× oil-immersion lens. The imaging was excited at 488 nm and emission monitored at 600–630 nm. All cells were washed with PBS before imaging. Microscopy was performed on a Leica TCS SP5 CLSM or a super-resolution microscopy Delta Vision OMX SIM respectively. Live cells were distinguished by their low To-Pro-3 emission with excitation at 633 nm and observed at 650–670 nm.

### Stimulated emission of depletion (STED) microscopy

Image acquisition of STED was obtained using a time gated STED (gSTED) microscope (Leica TCS SP8 STED 3X, Leica Microsystems, Germany) equipped with a HCX PL APO 100 × 1.40 NA oil objective. The image of [Ru(phen)_2_(dppz)]Cl_2_ was obtained using 488 nm excitation and 660/775 nm depletion. The detection wavelength range was set to 580–651 nm. The gated HyD detectors were set with the gated option on and the temporal gated selected was from 3.5 to 12 ns when depleting at 775 nm. For comparison, confocal images were acquired in the same field prior STED imaging. All images were obtained using the LAS AF software (Leica). Deconvolution processing was performed with Huygens Professional software (Scientific Volume Imaging, Hilversum, Netherlands).

We used fixed cells for chromosome imaging. After cell synchronization by double Thymidine block, cells fixed by 4% paraformaldehyde, then imaged by STED microscopy. And we used live cells for mitochondria imaging.

### 3D rendering of SIM images

[Ru(phen)_2_(dppz)]Cl_2_ and MTG renderings were created using the Spots tools and Surfaces tools respectively using IMARIS 9 software.

### 3D High-resolution microscopy and image analysis

3D images of HeLa cells were acquired on the DeltaVision OMX V3 imaging system (GE Healthcare) with a ×100/1.40 NA oil objective (Olympus UPlanSApo), solid-state multimode lasers (488, 405, 561 nm) and electron-multiplying CCD (charge-coupled device) cameras (Evolve 512 × 512, Photometrics). Serial Z-stack sectioning was done at 250 nm intervals for conventional mode. The microscope is routinely calibrated with 100 nm fluorescent spheres to calculate both the lateral and axial limits of image resolution. Conventional image stacks were processed by deconvolution methods using softWoRx 5.0 (GE Healthcare) with the following settings: Wiener filter enhancement 0.900; winner filter smoothing 0.800. The reconstructed images were further processed for maximum-intensity projections with softWoRx 6.1.1. Pixel registration was corrected to be less than 1 pixel for all channels using 100 nm Tetraspeck beads.

### Multi-SIM

Select the ‘Single Slice’ and ‘bright field’ imaging modes in the imaging software ‘VSIM’ of the Multi-SIM System, select the 488 nm laser, set the Laser to 33% (power of 50 mW), the exposure time is 5 × 10 ms, set the appropriate bright field light intensity, (for continuous cell irradiation: set the cycle count of the time series to 64, the cycle time to 1s, set the Focus), and finally perform image acquisition. After the data acquisition is completed, SIM reconstruction and Denoise processing are done in the reconstruction software ‘SIM_Image_Analyser’. The NA of the Multi-SIM lens is 1.49 (Nikon CFI SR HP Apo TIRF 100×/1.49 Oil objective lens).

### Nucleoid distribution and size measurement are performed using IMARIS 9.8 software

#### Nucleoid distribution

mtDNA were detected by the Imaris Spots utility, which finds spherical objects with a predefined radius (0.2 μm in the case of mtDNA). Nucleus surfaces were created using the Surfaces tools with manual editing using IMARIS. A center of mass function was used to extract the center of Nucleus. Then distances can be obtained from statistics table: ‘average distance of three nearest neighbors’ and ‘shortest distance to spots’.

#### Nucleoid size measurement

Nucleoid structures were segmented using the Surface segmentation utility available in Imaris, which detects objects based on local intensities. The resulting segmentation was visually inspected to remove small individual segmented objects components, which were regarded as noise. Then, equivalent diameter can be calculated through ‘number of voxels’ of surfaces.

### Agarose gel electrophoresis

The DNA agarose gel electrophoresis experiments were performed at room temperature. Increasing concentration of [Ru(phen)_2_(dppz)]Cl_2_ (0–100 μM) was added to 20 μl of Tris–HCl buffer with 5 μg/ml plasmid PBR322. After incubation for 30 min, all samples were placed in ice to end the reaction and then loaded onto 1% agarose gels containing Goldview and electrophoresed for 40 min at 80 V/cm.

### BSA, yeast RNA and ctDNA binding

Increasing concentration of DNA, RNA (0–25 μg/ml) or BSA (0–100 μM) was added to Tris–HCl buffer (10 mM, pH 7.4) with 10 μM [Ru(phen)_2_(dppz)]Cl_2_. The luminescence signal was measured after incubation at RT for 10 min.

### Photo-sensitizing effects *in vitro*

Cytotoxicity was analyzed in HeLa cells and determined by Alamar Blue assay as previously reported. Cells were incubated with 100 μM [Ru(phen)_2_(dppz)]Cl_2_ and 300 μM 2,3,4,5-TeCP for 0.5 h, washed with PBS for 3 times and incubated with fresh medium for 3 h. Then these cells were irradiated with a 450 nm LED light array (30 mW/cm^2^, 20 min). After another 24 h of incubation, cell viabilities of different groups were determined by Alamar Blue assay. Z-VAD-FMK (50 μM) added 1 h before irradiation were used for caspase inhibition.

### Cellular ROS detection

HeLa cells seeded into 6 well plates were treated with Ru at the indicated condition as mentioned above. Cells were stained with H_2_DCFDA (10 μM) for 20 min at 37°C in the dark and then irradiated with a 450 nm LED light array (30 mW/cm^2^, 20 min). Then cells were harvested and washed twice with serum-free DMEM. The fluorescence intensity of DCF in HeLa cells was measured by flow cytometry or imaged by CLSM.

### Caspase-3/7 activity assay

Caspase-3/7 activity was measured using the Caspase-Glo^®^ Assay kit (Promega, Madison, WI, USA) according to the manufacturer's instructions. Cells cultured in 96 well plates were treated with Ru. After irradiation (30 mW/cm^2^, 20 min), then incubated for another 24 h, 100 μl of Caspase Glo^®^ 3/7 reagent was added to each well containing 100 μl culture medium. The mixture was incubated at room temperature for 1 h and then luminescence was measured using a micro-plate reader.

## Results

### The formation and dissociation of [Ru(phen)_2_dppz]^2+^/2, 3,4,5-TeCP^−^ ion-pair complex results in selective delivery of [Ru(phen)_2_dppz]Cl_2_ in nucleus and mitochondria, respectively

[Ru(phen)_2_dppz]Cl_2_ was synthesized according to a previous study. Its chemical structure and ^1^H NMR spectrum is shown in Supplementary Figure S1. Firstly, the cellular distribution of [Ru(phen)_2_dppz]Cl_2_ after formation and dissociation of [Ru(phen)_2_dppz]^2+^/2,3,4,5-TeCP^−^ ion-pair complex were observed by CLSM. Cells stained with [Ru(phen)_2_dppz]Cl_2_ alone have almost no luminescence in cells (Supplementary Figure S2A). Incubation of HeLa cervical cancer cells with 100 μM [Ru(phen)_2_dppz]Cl_2_ and 300 μM 2,3,4,5-terachlorophenol (2,3,4,5-TeCP) showed that clear nuclear localization occurs with 30 min. ICP-MS results demonstrated that 2,3,4,5-TeCP facilitated the cellular uptake of [Ru(phen)_2_dppz]^2+^ (Supplementary Figure S2B). Interestingly, after washed cells with PBS (to remove 2,3,4,5-TeCP in the cell culture medium) and refilled with fresh medium, and after three hours, luminescence was observed from the cytoplasm but not the nuclear region. Because [Ru(phen)_2_dppz]Cl_2_ has a high binding affinity with double strand ctDNA (5.1 × 10^6^ M^−1^), and mitochondria contain mtDNA, we speculated that [Ru(phen)_2_dppz]^2+^ might get into mitochondria after effluxing out of nucleus. Then we co-stained [Ru(phen)_2_dppz]Cl_2_ with Mito-Tracker Green (MTG), which displays an emission spectrum that is well separated from that of [Ru(phen)_2_dppz]^2+^, this will allow two probes to be imaged separately.

**Figure 1.**
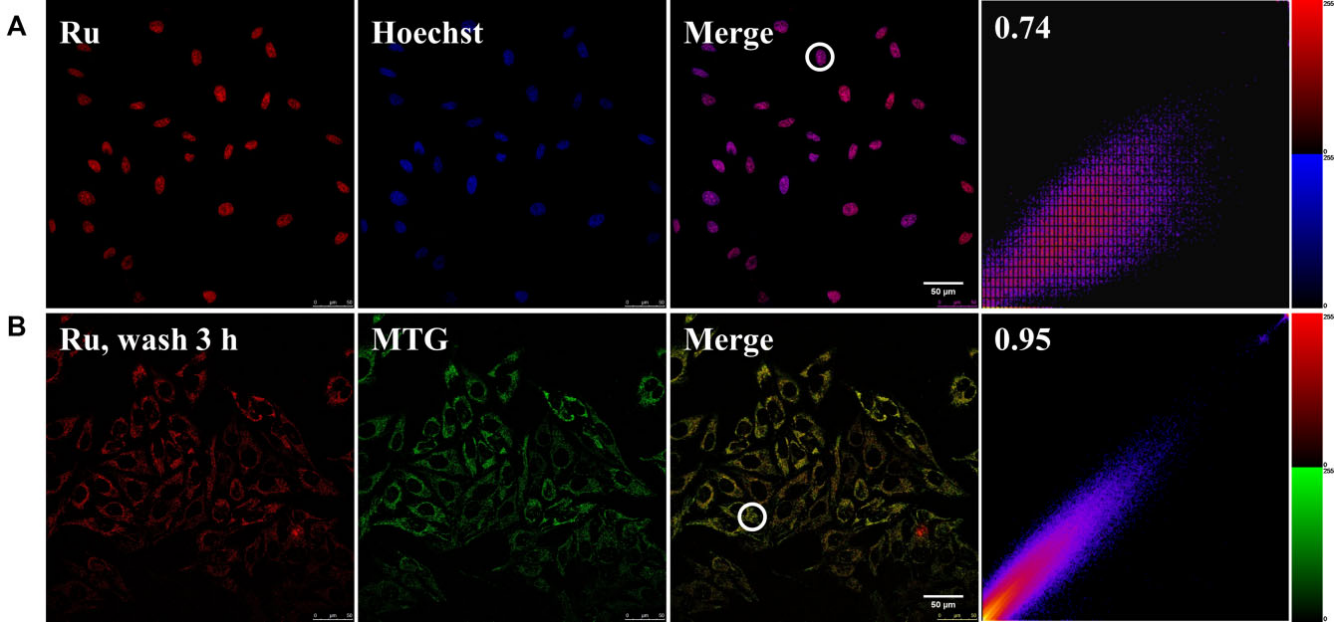
Representative CLSM images of cells stained with Hochest (blue), MTG (green) and co-stained with [Ru(phen)_2_dppz]^2+^ (red) under two different incubation conditions. (**A**) The luminescence was found to be mainly inside the nucleus when HeLa cells were incubated with [Ru(phen)_2_dppz]Cl_2_/2,3,4,5-TeCP for 30 min. (**B**) The luminescence was found to be mainly inside the mitochondria when HeLa cells were incubated with [Ru(phen)_2_dppz]Cl_2_/2,3,4,5-TeCP for 30 min, and then incubated with fresh medium for 3 h. The right two images showed Pearson's coefficients on the co-localization correlation intensity maps of the circles area in the merge image. Scale bar: 50 μm. Intensity scale: 0–255.

We carried out a detailed co-localization analysis when cells incubated with [Ru(phen)_2_dppz]Cl_2_ and then washed for 0 and 3 h. Probe overlap images clearly showed that after cells incubated with the complex for 0.5 h, the metal complex localizes in the nuclear DNA (Figure 1A). Incubation of HeLa cells with [Ru(phen)_2_dppz]Cl_2_ and co-staining with the DNA stain Hoechst shows strong co-localization of the probes. Conversely, co-staining with a nucleic acid dye SYTO 9 shows a clear difference in localization (Supplementary Figure S3A). Notably SYTO 9 preferentially binds RNA over DNA and as a consequence cytoplasm (the site of mRNA translating) and nucleoli (the site of rRNA synthesis and tRNA processing) are distinctly imaged through the stain's intense green emission. It is clear that [Ru(phen)_2_dppz]Cl_2_ is a DNA-specific stain as, in contrast to SYTO 9, no emission from the complex is observed from cytoplasm and nucleolar regions. We further confirmed that the Ru complex binds selectively to DNA rather than RNA by measuring the luminescence of the Ru complex when binding with DNA and RNA, its luminescence intensity is much weaker when bound to RNA than DNA (Supplementary Figure S3B, C). The emission of [Ru(phen)_2_dppz]^2+^ in cytoplasm after washed for 3 h was much brighter than that washed for 0 h (Supplementary Figure S4). And the confocal images and calculated Pearson's coefficients illustrate that strong co-localization between [Ru(phen)_2_dppz]^2+^ and MTG was observed when cells washed with PBS and incubated with fresh medium for 3 h (Figure 1B). Conversely, co-staining with Lyso-Tracker Green and ER-Tracker Green (as lysosome and endoplasmic reticulum stain, respectively), shows a clear difference in localization (Supplementary Figure S5). Since [Ru(phen)_3_]Cl_2_, [Ru(bpy)_3_]Cl_2_ and [Ru(bpy)_2_dppz]Cl_2_ have similar structure with [Ru(phen)_2_dppz]Cl_2_, these three Ru complex analogs were tested as mitochondria imaging agents. Firstly, cells incubated with different Ru complexes and chlorophenols, after Ru complexes distributed in nucleus, cells washed with PBS and incubated with fresh medium for 3 h. Similar to what we observed for [Ru(phen)_2_dppz]Cl_2_, the CLSM images illustrate that strong co-localization between these Ru complex analogs with MTG (Supplementary Figure S6). Interestingly, we found that [Ru(DIP)_2_dppz]Cl_2_ remains in nuclei even after washing the cells for 12 h, and resulted in the death of cells. [Ru(DIP)_2_dppz]Cl_2_ is known to be more lipophilic than the above mentioned Ru complexes, and it was found to bind with both proteins and DNA (60). Besides, [Ru(phen)_2_dppz]Cl_2_ was reported to efflux out of nuclei and cells by ATP binding cassette (ABC) transporter proteins (44), we speculated that [Ru(DIP)_2_dppz]Cl_2_ might bind with ATPase to inhibit the production of ATP, and subsequently affecting the function of ABC transporter proteins, which need to be studied in the future. Therefore, [Ru(DIP)_2_dppz]Cl_2_ is not suitable for mt-DNA imaging (Supplementary Figure S7).

In order to test whether [Ru(phen)_2_dppz]Cl_2_ alone can stain mitochondria or not, cells were incubated with high concentration of [Ru(phen)_2_dppz]Cl_2_ only (0.3 mM, without 2,3,4,5-TeCP) for 2 h. We found that [Ru(phen)_2_dppz]Cl_2_ can get into cytoplasm and distribute throughout cytoplasm under this condition, but interestingly, not concentrate in mitochondria (Supplementary Figure S8). Why [Ru(phen)_2_dppz]Cl_2_/TeCP co-treatment after washing but not by [Ru(phen)_2_dppz]Cl_2_ single treatment alone can make [Ru(phen)_2_dppz]Cl_2_ to selectively localize in mitochondria? Chemicals are difficult to penetrate into mitochondria because of the negative membrane potential and double membrane of mitochondria (61). Previous studies have shown that lipophilic, charged metal complexes and Ru complexes with a mitochondrial penetrating peptide can accumulate in the mitochondria (50,53). Highly lipophilic Ru complexes alone was found to get into cells through passive diffusion (62). Since [Ru(phen)_2_dppz]Cl_2_ neither contains a mitochondria targeted peptide nor has a high lipophilicity (lop *P* = −1.48) (60), it should be difficult to pass through mitochondria double membrane and might bind with other organelle in cytoplasm. [Ru(phen)_2_dppz]Cl_2_ is a kind of exogenous compound. When it binds with nuclear DNA, we expect that it will influence nuclear and cellular functions, resulting in cell death after long time incubation. Multidrug resistance (MDR) in cancer, which is defined as the resistance of cancer cells to antineoplastic agents by either intrinsic or acquired mechanisms (63). Many factors, such as efflux transporters, apoptosis regulation and DNA repair, are responsible for the development of MDR, and the most prominent one is associated with membrane ATP-binding cassette (ABC) transporters in cancer cells (64). We speculated that our washing with fresh medium will greatly reduce 2,3,4,5-TeCP (a typical uncoupler of oxidative phosphorylation) concentration, leading to increase of ATP production, thus activated ABC transporter proteins, and then triggered the efflux process. In order to verify this speculation, firstly, we tested the mitochondrial accumulation ability of [Ru(phen)_2_dppz]Cl_2_ in dead cells. [Ru(phen)_2_dppz]Cl_2_ accumulated in live but not fixed cellular mitochondria. It cannot efflux out of fixed cellular nucleus. This indicates that [Ru(phen)_2_dppz]Cl_2_ effluxed out of nucleus in an ATP dependent pathway (Supplementary Figure S9A). Secondly, we inhibited ABCB1, ABCC1, ABCG2 by the respective inhibitor PSC-833, Ko143 hydrate and probenecid. We can see clearly that [Ru(phen)_2_dppz]^2+^ remains accumulated in the nuclei in the presence of these inhibitors by CLSM, which indicate that these three specific inhibitors significantly prevented the efflux of [Ru(phen)_2_dppz]^2+^ from nuclei to mitochondria (Supplementary Figure S9B). Chlorophenols were reported to uncouple oxidative phosphorylation (65). When cells incubated with Ru complexes and chlorophenols together, chlorophenols can inhibit the efflux of Ru complexes by uncoupling oxidative phosphorylation, leading to inhibition of cellular ATP production. After cells washed with PBS and incubated with fresh medium, there has no sufficient chlorophenols to inhibit cellular ATP production, then [Ru(phen)_2_(dppz)]^2+^ can efflux out of nucleus and cells by the ATP-dependent ABC transporter proteins.

In short, the confocal microscopy results prove that, when cells stained with [Ru(phen)_2_dppz]Cl_2_ and 2,3,4,5-TeCP together, [Ru(phen)_2_dppz]^2+^ localizes in nucleus and binds to nuclear DNA, but when incubated in fresh medium for 3 h subsequently, [Ru(phen)_2_dppz]^2+^ efflux out of nucleus and re-localizes in mitochondria. These results also suggest that the formation and dissociation of ion-pair complex can influence the cellular distribution of [Ru(phen)_2_dppz]Cl_2_.

### High resolution SIM imaging demonstrated the mitochondrial DNA staining by the Ru(II) complex

**Figure 2.**
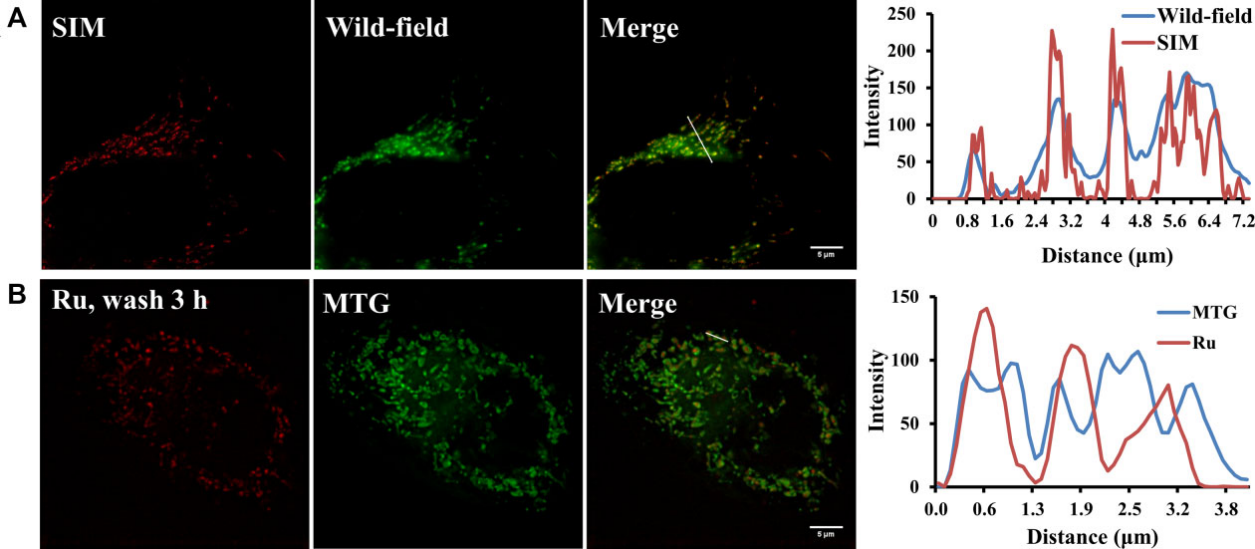
SR SIM and 3D high resolution imaging of mitochondria by [Ru(phen)_2_dppz]Cl_2_ via dissociation of ion-pairing complex with chlorophenolate counter-anions. (**A**) Wide-field microscope (WF) and SIM images of HeLa cells stained with [Ru(phen)_2_dppz]Cl_2_. Luminescence intensity profile plots along white line of the images, showed improved resolution in SIM. (**B**) 3D high resolution images of HeLa cells stained with [Ru(phen)_2_dppz]Cl_2_. Co-staining [Ru(phen)_2_dppz]Cl_2_ with mitochondrial stain MTG. Scale bar: 5 μm.

**Figure 3.**
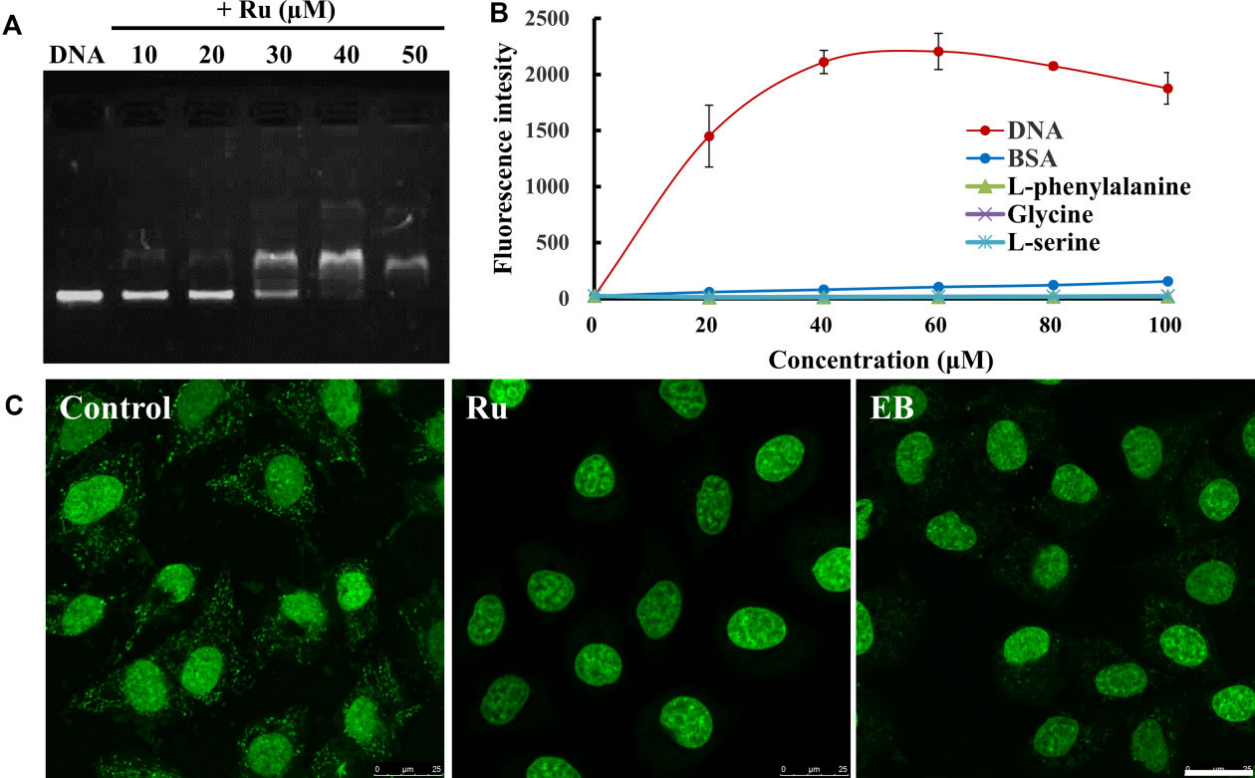
Evidence for the preferential binding of [Ru(phen)_2_dppz]Cl_2_ with live-cell mtDNA. (**A**) Agarose gel electrophoresis experimental evidence for the binding of Ru (50 μM) with plasmid DNA. The migration of plasmid PBR322 DNA was gradually inhibited by adding increasing concentration of Ru. (**B**) Luminescence intensity of Ru (10 mM Tris–HCl, pH 7.4) in the presence of BSA and three typical amino acids (l-phenylalanine, glycine and l-serine) up to 100 μM compared to their luminescence intensity when saturated with ctDNA. Data represent mean ± S.D. of three independent experiments. (**C**) Quenching of PicoGreen (3 μl/ml) fluorescence by Ru complex (cells incubated with 0.1 mM Ru and 0.3 mM 2,3,4,5-TeCP for 0.5 h and then incubated with fresh medium for 3 h) and EB (1.0 μg/ml, 1.5 h) in HeLa cells. Scale bar: 25 μm

Having established that [Ru(phen)_2_dppz]Cl_2_ is an ion-pair formation and dissociation dependent dye for both nuclear and mitochondrial DNA by confocal microscopy, we investigated its potential application as a super resolution probe for SIM. The Ru complex generated striking SIM images of mitochondria, with the improved spatial resolution clearly displayed in a comparative luminescence intensity profile plots (Figure 2A). The low photo-bleaching and bright mitochondria/nucleus localized luminescence of [Ru(phen)_2_dppz]Cl_2_ is ideally suited for SIM imaging, revealing details of mitochondria structure. Then we co-stained [Ru(phen)_2_dppz]Cl_2_ with MTG and imaged by 3D high-resolution microscopy to further analyze its mitochondrial localization. From luminescence intensity profile plots along three mitochondria of the merge image of Ru with MTG (Figure 2B), Z slice images (for dynamic process, see Supplementary Video S1) and volume view videos (for dynamic process, see Supplementary Video S2), we can see the emission of MTG centered around the emission of Ru, which indicate that they localized in different parts of the mitochondrion. MitoTracker dyes are based on location of lipophilic cations in the inner mitochondrial membrane which is highly rippled/tortuous. In clear contrast, since [Ru(phen)_2_dppz]^2+^ has DNA ‘light-switching’ effect (it has no emission in water, but has strong emission when binding with DNA), the stronger emission from the center of mitochondria was most probably due to its binding to mitochondrial DNA. Although [Ru(phen)_2_dppz]^2+^ can also bind to other surrounding biomolecules, the imaged [Ru(phen)_2_dppz]^2+^ emission is mainly came from mtDNA binding because its luminescence intensity is typically much weaker when bound to protein or RNA (Figure 3B) (66).

Since mtDNA is a kind of plasmid DNA, which is a double strand circular DNA, we tested the binding ability of the Ru complex with commonly used plasmid DNA PBR322 by agarose gel electrophoresis. As we know, due to the large number of negative charges on DNA, DNA would migrate from negative electrode to positive electrode when it was subjected to electrophoresis. The migration should be inhibited if the negative charges of DNA were compromised by the positive-charges of [Ru(phen)_2_(dppz)]^2+^. Indeed, we found that the migration of plasmid PBR322 DNA was gradually inhibited by adding increasing concentration of [Ru(phen)_2_dppz]Cl_2_ (0–100 μM), which indicated the binding of the Ru complex with the plasmid DNA (Figure 3A).

We further verified the live cell mtDNA binding ability of [Ru(phen)_2_dppz]Cl_2_ by a classical mtDNA intercalator PicoGreen (Figure 3C). After adding another intercalating dye, the fluorescence of PicoGreen will be quenched because of the intercalator's competitive binding with mtDNA. Cells stained by PicoGreen had nuclear fluorescence along with many punctate mtDNA fluorescence in cytoplasm. In contrast, the addition of [Ru(phen)_2_dppz]Cl_2_ results in the complete quenching of mtDNA nucleoid staining, which is more obvious than that observed for the well-established mtDNA intercalator EB.

**Figure 4.**
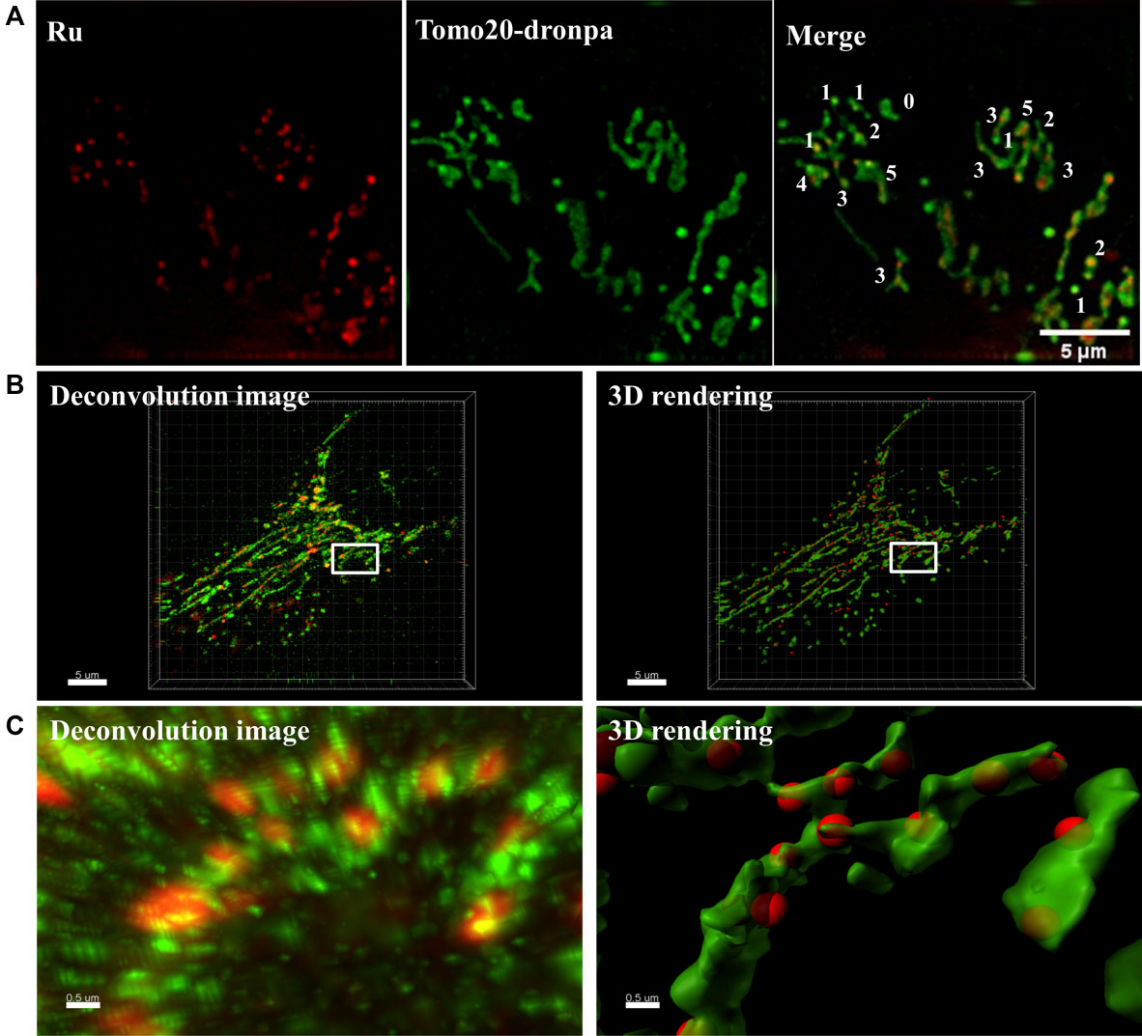
Evidence for preferential binding of [Ru(phen)_2_dppz]Cl_2_ with live-cell mtDNA. (**A**) Representative deconvolution images of Ru and Tomo20-dronpa in a single cell showed mtDNA stained by Ru (red spots) and the mitochondrial membrane stained by Tomo20-dronpa. White numbers indicated the number of nucleoids in a mitochondrion. Scale bar: 5 μm. (**B**, **C**) 3D High-resolution microscopy and 3D rendering images of Ru and MTG. Higher magnification images (C, scale bar: 0.5 μm) corresponding to the box in the lower magnification deconvolution and 3D rendering images (B, scale bar: 5 μm). Left: 3D SIM images produced by staining HeLa cells with Ru (red color) and MTG (green color). Right: The red spots indicate the mtDNA stained by Ru, the green strips indicate the mitochondrial membrane stained by MTG.

Then, we observed clearly the mtDNA binding of [Ru(phen)_2_dppz]Cl_2_ by two following experiments. Firstly, in order to see more clearly the localization of Ru and MTG, we got 3D images of mitochondria stained with these two dyes and created 3D rendering of these two dyes. We found that the emission of Ru complex looks like peas inside MTG pea hulls (Figure 4B, C). Secondly, we co-stained Ru complex with recombinant human translocase of outer mitochondrial membrane 20 tagged with dronpa fluorescent protein (tomo20-dronpa). When cells incubated with [Ru(phen)_2_dppz]Cl_2_/2,3,4,5-TeCP for 30 min, Ru complex localized mainly in nuclei and has no emission in mitochondria (Supplementary Figure S10A). Then incubating cells with fresh medium for another 3 h, the emission from nuclei disappeared and the almost complete overlay images clearly display the mitochondria stain of Ru complex (Supplementary Figure S10B). Furthermore, 3D rendering images with higher resolution clearly showed that tomo20-dronpa stains mitochondrial membrane and the emission of Ru complex appears like punctate spots in the mitochondria, which indicates the mitochondrial DNA stained by Ru complex (Figure 4A).

### Time gated STED imaging of nuclear and mitochondrial DNA

The high resolution of SIM images over long imaging time re-affirmed the photo-stability of the probe. Since Ru complex has long emission lifetime, which increases the probability of stimulated emission, and it shows a large Stoke shift, which provides ‘spectral width’ for excitation, emission and depletion, the application of [Ru(phen)_2_dppz]Cl_2_ as a STED probe was then studied. Time gated STED (gSTED) can not only increase image resolution, but also decrease laser intensity, which avoid photo-bleaching and photo-toxicity to fluorescent samples. In the STED microscopy technique, although the phosphor at the edge of the excited spot would be caused stimulated emission by high intensity depletion light, a small number of electrons would inevitably return to the ground state by means of auto-fluorescence. The fluorescence lifetime changes in the presence of depleting light: the higher the depletion light intensity, the shorter the fluorescence lifetime. Therefore, the auto-fluorescence lifetime at the edge of the excited spot is smaller than that at the center of the excited spot. Based on this principle, gSTED eliminated the influence of short lifetime auto-fluorescence at the edge of excited spot by time-delay detection (fluorescence is detected at intervals after excitation light action), and therefore improved the resolution of the system.

**Figure 5.**
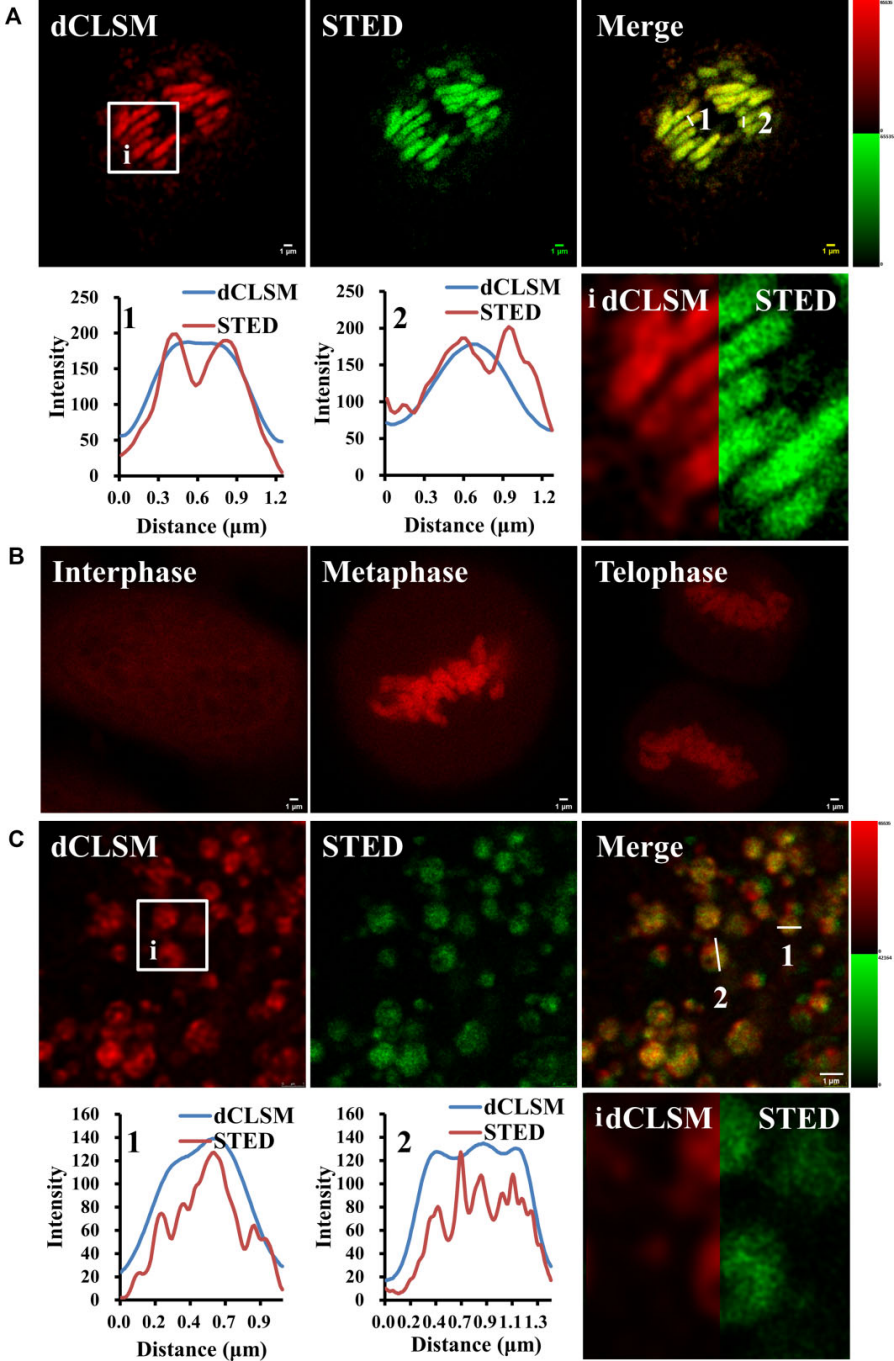
Super-resolution STED imaging of nuclear and mitochondrial DNA by [Ru(phen)_2_dppz]Cl_2_. (**A**) Deconvoluted CLSM (dCLSM) and STED images of chromosomes stained by the Ru complex during metaphase. (**B**) STED images of nuclear DNA in fixed HeLa cells stained by Ru complex during the cell cycle process. (**C**) Deconvoluted CLSM and STED images of mitochondrial DNA stained by Ru complex. White squared insets in A and C are magnified in (i-ii). Enhancement of the resolution by STED can be observed in the compared dCLSM/STED images. Line traces through single chromosomes (1) and (2) and single live cellular mitochondria (3 )and (4) and the corresponding plot profiles show greatly improved resolution of STED images compared to deconvoluted CLSM. The STED images were obtained using 775 nm depletion (A, C) and 660 nm depletion (B). Pixel size for recording STED images was 0.019 μm (A, B) and 0.011 μm (C). Scale bar: 1 μm. Intensity scale: 0–65535 except for the STED image in C: 0–42164.

Firstly, we applied 660 and 775 nm beam to deplete the luminescence of the Ru complex. We successfully obtained STED images in both set of conditions, and after incubating the cells with [Ru(phen)_2_dppz]Cl_2_/2,3,4,5-TeCP for 30 min, super resolution imaging of chromatin could be accomplished (Figure 5A). The improvement in STED image resolution is significant and sister chromatids can be distinguished. A line profile drawn through a single chromosome reflected the relative improvement of the STED image, where with STED, but not CLSM, each chromatid is resolved. The DNA ‘light-switch’ property of [Ru(phen)_2_dppz]Cl_2_ together with STED allows facile recognition of cell cycle phase through nuclear STED images of labeled HeLa cell during the cell cycle process (Figure 5B). The chromosomes replicate at interphase, line up along the metaphase plate of the cell at metaphase, separated chromatid being pulled toward the pole in anaphase and the chromosomes begin to decondense during telophase are all clearly distinguishable with outstanding clarity. Therefore, [Ru(phen)_2_dppz]Cl_2_ is evidently well suited for STED. On the other hand, when incubated cells with fresh medium for 3 h after nuclear imaging, [Ru(phen)_2_dppz]Cl_2_ produced super resolution STED images of mitochondria, with the improved resolution clearly shown in a comparative intensity profile (Figure 5C).

### Each mitochondrion containing 1-8 mtDNA molecules were found to be distributed throughout the mitochondrial matrix in live cells using this new mtDNA probe

Mitochondrion is a necessary organelle that plays a vital role in fundamental cellular processes such as calcium signaling, apoptosis and oxidative phosphorylation. They contain mitochondrial DNA, which encode a handful of rRNAs and tRNAs necessary for intra-mitochondrial translation as well as essential mitochondrial proteins. In this study, we revealed the organization of mtDNA in live HeLa cells. Mitochondrial DNA was counted manually on images captured by 3D high-resolution microscopy of conventional mode. The high mitochondrial luminescence allowed easy identification and counting of nucleoids in HeLa cells. The total number of nucleoids was thus determined after the merge of five focal planes (covering the entire cell volume) to a single image. Using [Ru(phen)_2_dppz]Cl_2_ as mtDNA stain and Tomo20-dronpa for whole mitochondria stain, we showed that nucleoids containing 1–8 mtDNA molecules are distributed throughout the entire mitochondrial matrix (Supplementary Figure S11).

### Nucleoid distribution and size

**Figure 6.**
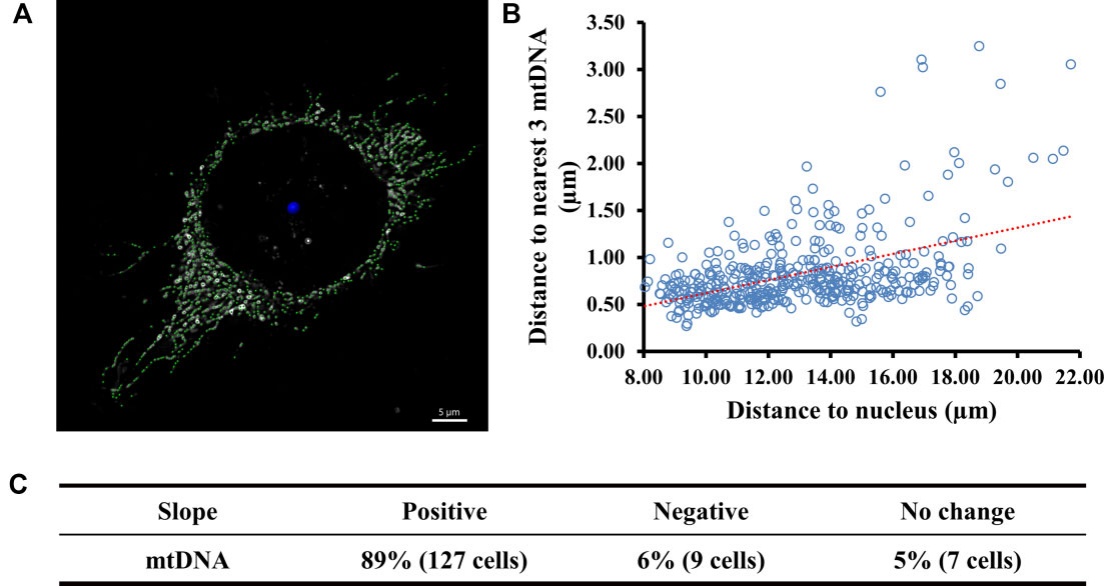
Nucleoid distribution as a function of the distance to the nucleus. (**A**) Representative cell labeled with [Ru(phen)_2_dppz]Cl_2_ for mtDNA used for the analysis presented in (B). The green spots denote mitochondria, the bigger blue spot indicates the center of the cell nucleus. Scale bar: 5 μm. (**B**) Plot of the average distance to three nearest neighbors of individual nucleoids as depending of the shortest distance to the center of nucleus. The blue circles represent 406 nucleoids of a cell. (**C**) Table summarizing the analysis of altogether 143 cells as exemplified in (A) and (B).

In order to find out whether the distribution of nucleoids varies with the distance to the nucleus, we determined for each nucleoid the average distance to its nearest 3 neighbors as a function of the distance to the nucleus center of 143 randomly selected cells in multi-SIM images. We observed an increase in the nearest neighbor distances when comparing perinuclear nucleoids to those in the cell periphery in 89% of the [Ru(phen)_2_dppz]Cl_2_ labeled cells (n = 127 cells) (Figure 6). These results suggest that there are more proximal nucleoids in live HeLa cells, which in agreement with previously reported DNA/TFAM antibody-labeled immunofluorescence method in fixed cells (67). Mitochondrion is a semi-autonomous organelle, the majority of the mitochondrial genes, including polymerase and transcriptional cofactors, are nuclear-encoded (61). We speculated that shorter distance to nucleus facilitate transportation of nucleus-encoded proteins to mitochondria. Additionally, nucleus is one of the most important organelles where DNA replication and transcription take place, which need abundance of ATP, and mitochondrion is the ATP production organelle. The shorter distance of mitochondria to nucleus may ensure the nucleus has an adequate supply of ATP.

The nucleoids mean size stained with [Ru(phen)_2_dppz]Cl_2_ was calculated in live HeLa cells. Multi-SIM imaging of mitochondrial nucleoids visualized by [Ru(phen)_2_dppz]Cl_2_ results in a punctuate pattern within the mitochondrial network of HeLa cells. mtDNA structures were segmented using the Surface segmentation utility available in Imaris, which detects objects based on local intensities. 46 274 mtDNA from 144 cells were picked out by this method. The calculated data from multi-SIM images revealed that the nucleoids have an average diameter of 310 nm (120–757 nm) (Supplementary Figure S12). Since some mtDNA distribute very close to each other, which cannot be picked apart by the software, we think the actual mtDNA diameter is smaller than the average 310 nm and closer to the minimum 120 nm. Previous TEM studies reported sizes of ∼70 nm of immunogold-labeled single nucleoids; (68) and STED microscopy in conjunction with three different markers (DNA antibodies, TFAM antibodies, and immune-detection of incorporated BrdU) gave a mean nucleoid size of ∼100 nm (67). The mean nucleoid size measured here by multi-SIM is bigger than the above-mentioned two methods possibly because the diffraction limit of SIM is lower than that of STED and TEM imaging. But TEM sample preparation is complicated and STED is more suitable for fixed cells since it need longer imaging time and stronger laser power than SIM. Multi-SIM imaging of mtDNA stained by the Ru complex has its unique advantages, such as suitable for live cell imaging, long time imaging and abundant mtDNA imaging.

### Enantioselective imaging of live-cell mtDNA was observed between the two chiral forms (Δ- and Λ-enantiomer) of the Ru(II) complex

**Figure 7.**
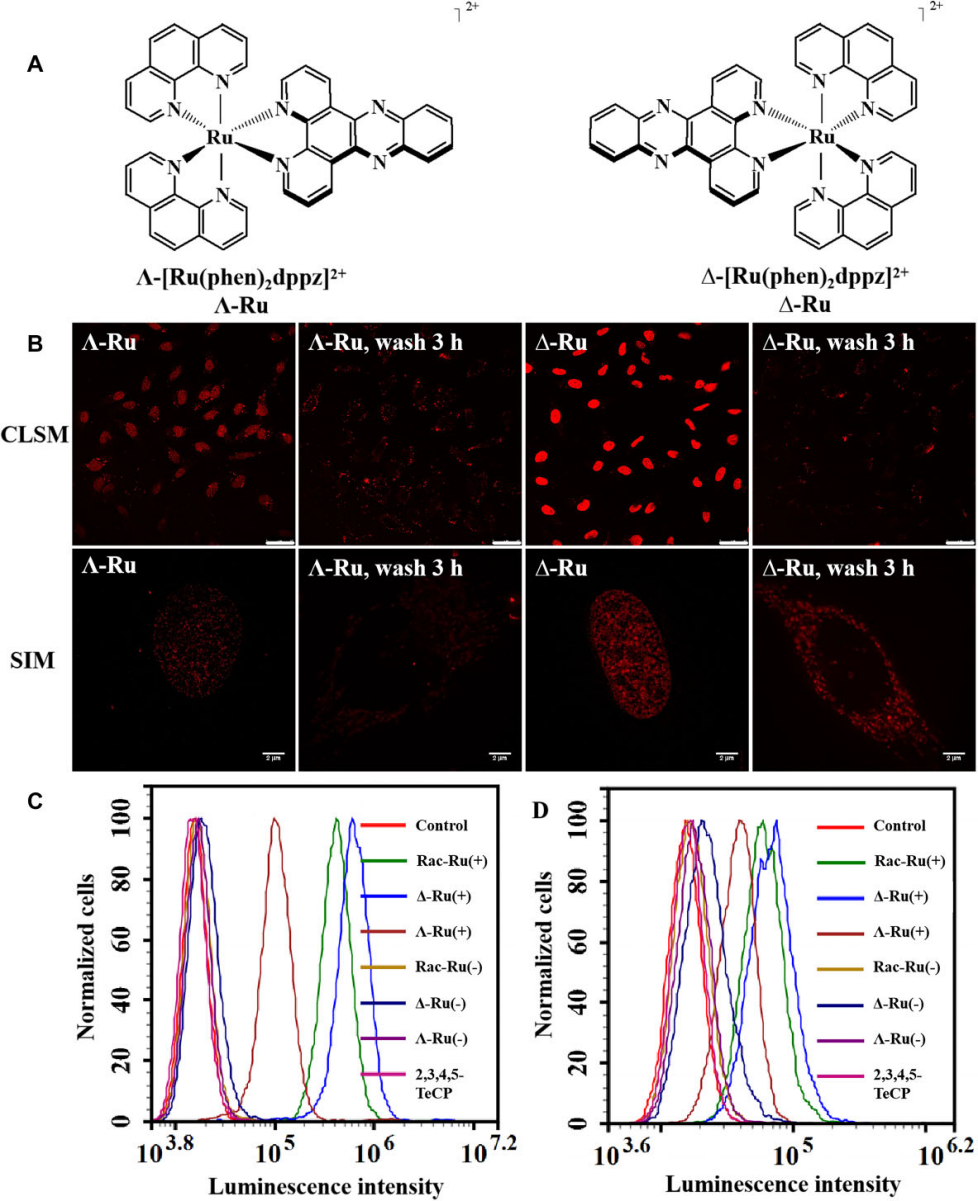
Enantio-selective live-cell mitochondrial DNA targeting and super-resolution imaging by the two enantiomers of the chiral Ru complex. (**A**) Chemical structures of two chiral forms of Ru complex. B and C: CLSM/SIM images (**B**) and FACS intensity (**C**) of cells stained by Δ-Ru/Λ-Ru (100 μM) with 2,3,4,5-TeCP (300 μM) for 30 min (for nuclei staining) (**C**), and then washed with PBS and incubated for another 3 h (for mitochondria staining) (**D**). Scale bar (CLSM): 50 μm. Scale bar (SIM): 2 μm. (+): incubated with 2,3,4,5-TeCP, (−): incubated without 2,3,4,5-TeCP.

The above studies employed racemic Ru(II) complex. The use of chiral forms of metal complexes to probe DNA structure are becoming of increasing interest. The chiral forms of Ru(II)–dppz complexes have been successfully applied to distinguish left- and right-handed duplex DNA (69–72) So the chirality of Ru(II) complex should be an important characteristic for an intracellular DNA structural probe (Figure 7A). The two enantiomers of [Ru(phen)_2_(dppz)]Cl_2_ were separated by high-performance liquid chromatography. The Δ-enantiomer eluted first, followed by the Λ-isomer (Supplementary Figure S13A). Assignment of the two fractions was confirmed by circular dichroism (Supplementary Figure S13B). Interestingly, from CLSM images and SIM images with higher resolution, we can see that the Δ-enantiomer of Ru(II) complex displayed much higher luminescence intensity not only in the nucleus, but also in the mitochondria than the Λ-enantiomer (Figure 7B). The luminescence intensity of Δ-enantiomer in nucleus (Figure 7C) and mitochondria (Figure 7D) is about 5.60 and 1.98 times higher than that of Λ-enantiomer, respectively. The original intuition that the Δ-enantiomer of octahedral Ru(II)-dppz complex must be a better fit for the right-handed DNA duplex is also visualized here for the first time in live-cell mitochondria. It was reported that the binding affinity of Δ-enantiomer of Ru(II) complex with ctDNA (3.2 × 10^6^ M^−1^) was bigger than that of the Λ-enantiomer (1.7 × 10^6^ M^−1^). Therefore, the higher quantum yield, stronger binding affinity and longer excited-state lifetimes of the Δ-enantiomer bound to DNA together might generate its brighter luminescence intensity than the Λ-enantiomer (Supplementary Figure S13C) (73). In order to investigate the detection limit of the chiral and racemic forms of Ru(II)-dppz complexes applied in live-cell mtDNA SRM, cells incubated with different concentrations of chiral and racemic Ru(II) complexes, then imaged by SIM. From SIM images, we can see that cellular mitochondria can be observed by as low as 25 μM Ru(II) complexes (Supplementary Figure S14).

### Enantioselective apoptotic cell death was induced for cells pretreated with the two enantiomers of [Ru(phen)_2_(dppz)]Cl_2_/2,3,4,5-TeCP upon prolonged visible-light irradiation

As we described above, [Ru(phen)_2_(dppz)]Cl_2_ entered into mitochondria and bind with mtDNA after dissociation of ion-pair complex with 2,3,4,5-TeCP. Since Ru-dppz complexes are reported to be photo-activated to form its excited states, which can cause DNA damage either by direct oxidation or by forming ROS, we expect that mtDNA damage and cell apoptosis should be induced for cells when [Ru(phen)_2_(dppz)]Cl_2_ accumulated in mitochondria with prolonged light irradiation.

It has been demonstrated that some DNA intercalating drugs such as doxorubicin and ethidium bromide can induce mtDNA remodeling. Since [Ru(phen)_2_(dppz)]Cl_2_ is also a DNA intercalator, so we want to know whether it can induce mtDNA remodeling or not. Interestingly, our new experimental results showed that nucleoid remodeling/mtDNA damage and cell apoptosis could be induced in mitochondria accumulated [Ru(phen)_2_(dppz)]Cl_2_ after prolonged light irradiation. PicoGreen is a DNA-intercalating fluorophore that can label mtDNA. The bright fluorescence of PicoGreen is almost quenched in cellular mitochondria when [Ru(phen)_2_(dppz)]Cl_2_ accumulated in mitochondria. After visible light (450 nm) irradiation, the formation of bright and giant nucleoids (arrows in Supplementary Figure S15A) are observed in racemic and chiral [Ru(phen)_2_(dppz)]Cl_2_ stained cells, which is known as nucleoid remodeling. The average nucleoid diameter of these enlarged nucleoids was 1–3 μm, compared to ∼0.6 μm of normal sized nucleoids in CLSM images (Supplementary Figure S15B). Different from previously reported intercalators, which induced mtDNA remodeling without irradiation, we found that irradiation of mtDNA intercalated [Ru(phen)_2_(dppz)]Cl_2_ is required to induce mtDNA remodeling.

It is known that mitochondria are the main intracellular source of ROS production and mtDNA damage usually results in the elevation of ROS. We initially detected intracellular ROS levels in Ru complex stained cells by 2',7'-dichlorodihydrofluorescein diacetate (H_2_DCFDA) staining and flow cytometry. After treatment, a dramatic ROS elevation is observed in Ru complex treated cells after illumination. The mean fluorescence intensity in cells treated with racemic [Ru(phen)_2_(dppz)]Cl_2_, Δ-enantiomer and Λ-enantiomer increased by approximately 92.5-, 112.2- and 80.8-fold, respectively (Supplementary Figure S16B). Moreover, co-localization experiments showed that although ROS are generated in whole cells, they mainly generated in mitochondria, as indicated by the overlap between the fluorescence of DCF and Mito-Tracker Deep Red (MTDR) (Supplementary Figure S16A, S17C, D).

**Figure 8.**
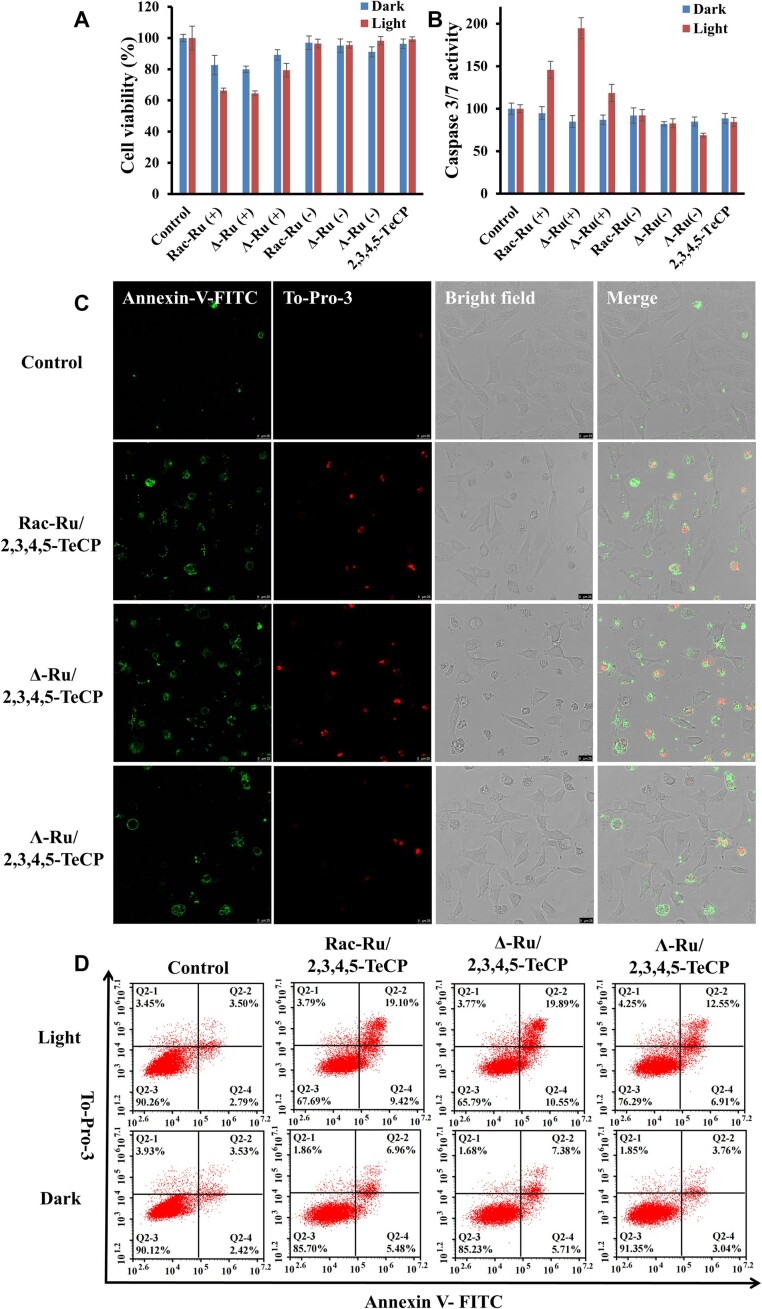
Enantioselective induction of apoptosis in cells pretreated with [Ru(phen)_2_(dppz)]Cl_2_/2,3,4,5-TeCP after prolonged visible light irradiation. After treatment with Ru (100 μM)/2,3,4,5-TeCP (300 μM) for 0.5 h, cells were rinsed with PBS for 3 times, refilled with new fresh medium and incubated for 3 h, then cells were exposed to light irradiation (450 nm, 30 mW/cm^2^, 20 min). (**A**) Cell viability were tested by Alamar Blue assay after incubation for another 24 h. (**B**) Detection of caspase-3/7 activity in HeLa cells treated with Ru in the absence or presence of light. Data represent the mean ± SD of 5 independent experiments. (C and D) Cells were stained with Annexin V-FITC (10 μM) and To-Pro-3 (0.5 μM) for 15 min after incubation for another 24 h, then imaged by CLSM (**C**) and detected by FACS (**D**). Scale bar: 25 μm.

The cytotoxicity induced by [Ru(phen)_2_(dppz)]Cl_2_ either in the dark or under irradiation were evaluated in HeLa cells by Alamar Blue assay (Figure 8A). [Ru(phen)_2_(dppz)]Cl_2_ alone showed no cytotoxicity both in the dark and under irradiation, which is reasonable since [Ru(phen)_2_(dppz)]Cl_2_ alone almost cannot get into cells (Supplementary Figure S2). Racemic and the two enantiomers of [Ru(phen)_2_(dppz)]Cl_2_ incubated with 2,3,4,5-TeCP showed low cytotoxicity in the dark. Upon 450 nm visible light irradiation, the phototoxicity of mitochondria accumulated [Ru(phen)_2_(dppz)]Cl_2_ increased, which indicates that it should be suitable for photo-dynamic therapy (PDT) applications. Both the dark cytotoxicity and phototoxicity of mitochondria accumulated Δ-enantiomer was found to be higher than that of mitochondria accumulated Λ-enantiomer. The viability of cells treated with Δ-enantiomer/2,3,4,5-TeCP after irradiation is 64.5%, which is much lower than 79.4% of Λ-enantiomer/2,3,4,5-TeCP.

Mitochondria were known to mediate essential cell functions such as apoptosis. Thus, we further investigated whether the Ru complex in mitochondria after light irradiation can induce apoptosis or not. Indeed, programmed cellular death was observed for such cells after continuous visible light-activation, as demonstrated by Annexin V- FITC/To-Pro-3 assay (Figure 8C, D, Supplementary Figures S17A, B, S18). Apoptotic cells increased significantly upon light-activation of mitochondria accumulated Ru complex. An approximately 2.3-, 1.5- and 1.4-fold increases in caspase-3/7 activity were observed in cells treated with Δ-, racemic or Λ-[Ru(phen)_2_(dppz)]Cl_2_ upon irradiation, respectively (Figure 8B). Furthermore, Z-VAD-FMK, a pan-caspase inhibitor, can efficiently inhibit cell death caused by Ru-mediated PDT (Supplementary Figure S19). These results indicate that mitochondria accumulated [Ru(phen)_2_(dppz)]Cl_2_ mediated photo-dynamic effects induced cell death through caspase-dependent apoptosis.

We firstly speculated that the enantio-selective induction of cell apoptosis might be related to their different cellular uptake efficacy. However, cellular even nuclear uptake efficacy of the two chiral Ru complexes was found to be identical as measured by ICP-MS in our previous study (44). So, the enantio-selective induction of cell apoptosis was not caused by different cellular uptake efficacy of the two enantiomers in this study. Since Ru complex binds with mtDNA in mitochondria, we then speculated that the enantio-selective induction of cell apoptosis might be related to their different binding affinity and binding geometry with DNA. Firstly, the binding affinity of Δ-enantiomer is 1.9 times stronger than that of the Λ-enantiomer (for Δ-enantiomer, *K* = 3.2 × 10^6^ M^−1^, while for Λ-enantiomer, *K* = 1.7 × 10^6^ M^−1^) (74). Secondly, the relative quantum yield and excited state lifetimes of the DNA-bound Λ-enantiomer is significantly lower than the DNA-bound Δ-enantiomer, which is due to the poorer fit of the left-handed Ru complex into the right-handed DNA helix, making the phenazine nitrogens of the dppz ligand more accessible to water (75,76). Because of the better geometry fit and higher binding affinity of Δ-enantiomer with right-handed DNA, the ROS produced by Δ-enantiomer during illumination may attack DNA more effectively and thus caused more cellular apoptosis. As we know, mitochondrion has been a sensitive and effective target for inducing cell death. Therefore, these results further confirm that mtDNA is the main target of [Ru(phen)_2_(dppz)]Cl_2_ complex in inducing cell damage, and the Δ-enantiomer played a dominating role in killing cells in photosensitizing by its better geometry fit and higher binding affinity than that of Δ-enantiomer with the right-handed mtDNA.

In short, the enantio-selective induction of cell apoptosis and mtDNA damage were observed for the two chiral form (Δ- and Λ-) of [Ru(phen)_2_(dppz)]Cl_2_ complex upon visible light irradiation, with Δ-enantiomer showing much stronger effects compared with Λ-enantiomer. We speculated that the cellular photo-toxicity was induced by ROS (probably ^1^O_2_), which was produced by [Ru(phen)_2_(dppz)]Cl_2_ (42,77). As we know, the diffusion distance and lifetime of ROS is short. Therefore, these results further confirm that [Ru(phen)_2_(dppz)]Cl_2_ complex not only need to reach the target organelle mitochondria, but also the Δ-enantiomer played a dominating role in inducing cell apoptosis via photo-sensitizing by a better binding geometry and stronger binding affinity with mtDNA.

### In-situ self-monitoring apoptosis process by mtDNA-targeted [Ru(phen)_2_(dppz)]Cl_2_

Although PDT thrived as a promising treatment, highly active photosensitizers and intense light power can cause treatment overdose. However, extra therapeutic response probes always make the monitoring process complicated, ex-situ and delayed. Now this challenge is addressed by the mtDNA-targeted [Ru(phen)_2_dppz]Cl_2_ facilitated by 2,3,4,5-TeCP. We found, unexpectedly, that [Ru(phen)_2_dppz]Cl_2_ undergoes mitochondria-to-nucleus translocation during apoptosis induced by visible light irradiation, thus enabling the in-situ self-monitoring via fluorescence migration.

When [Ru(phen)_2_(dppz)]Cl_2_ got into mitochondria, we imaged cells by continuous 488 nm laser excitation to see whether we can observe some new and unique biological effects or not. At the beginning, [Ru(phen)_2_(dppz)]Cl_2_ stained the mitochondria exclusively. However, after 10 min light illumination, [Ru(phen)_2_(dppz)]Cl_2_ redistributed mainly in nucleus, while the area of mitochondria seemed fragmented and showed negligible fluorescent signal. Furthermore, cell shrinkage, membrane blebbing and morphology change can be visualized in the bright field images, which are the traits of cell apoptosis (Supplementary Figure S20, for dynamic process, see Supplementary Video S3). These findings suggested that mitochondria-to-nucleus translocation of [Ru(phen)_2_dppz]Cl_2_ took place during cell apoptosis.

**Figure 9.**
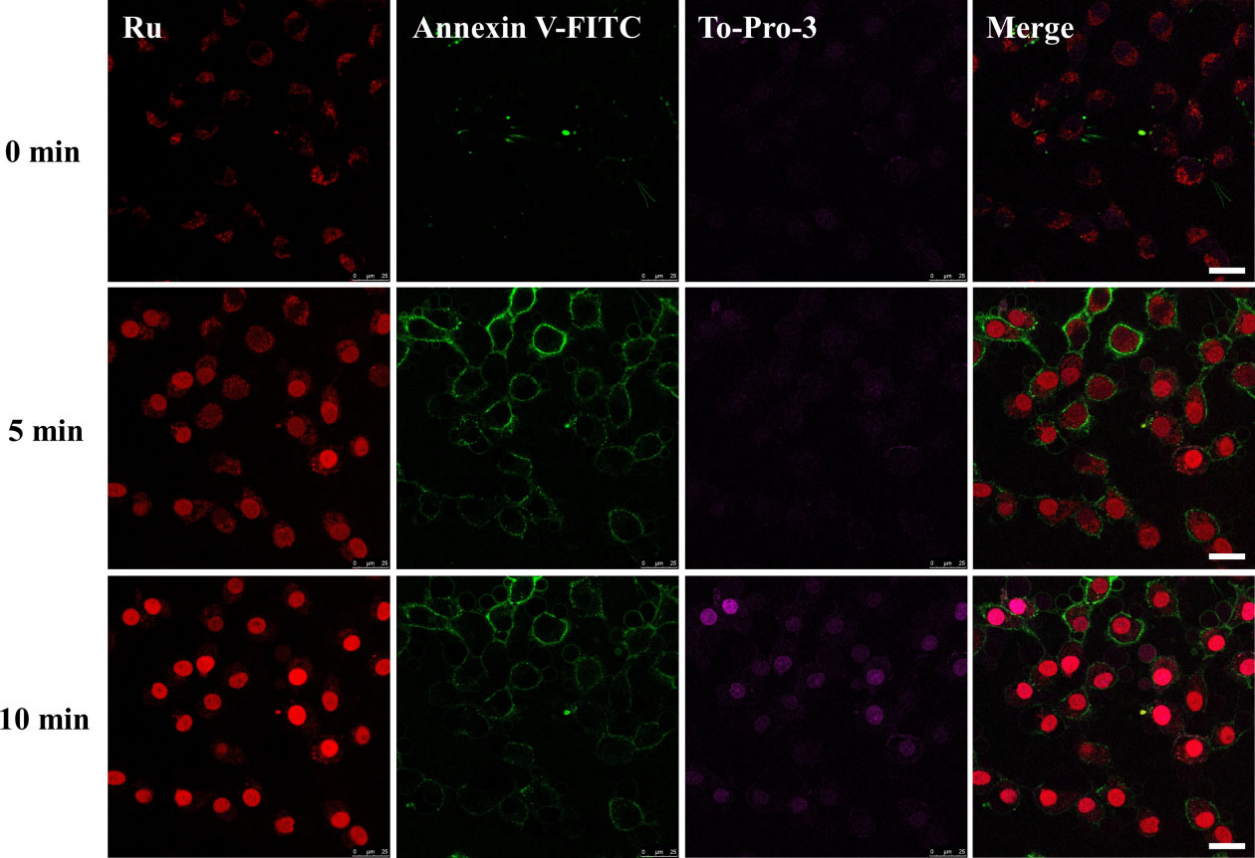
In-situ self-monitoring apoptosis process by mitochondria accumulated [Ru(phen)_2_(dppz)]Cl_2._ Real-time CLSM imaging of HeLa Cells under continuous visible light irradiation stained with [Ru(phen)_2_(dppz)]Cl_2_, Annexin V-FITC and To-Pro 3. Scale bar: 25 μm.

In order to uncover the mechanism of luminescence translocation phenomenon, cells were stained with Annexin V-FITC and To-Pro-3 to distinguish early/late stage apoptosis from necrosis. After [Ru(phen)_2_dppz]Cl_2_ accumulated in mitochondria, HeLa cells were stained with Annexin V-FITC and To-Pro-3, followed by visible light irradiation to induce cell death (Figure 9). [Ru(phen)_2_dppz]Cl_2_ was found to be accumulated in mitochondria of live cells at first. After continuous 5 min visible light irradiation, the luminescence of [Ru(phen)_2_dppz]Cl_2_ in mitochondria decreased while that in nucleus increased. At the same time, green fluorescence of Annexin V-FITC emerged in cell membrane while To-Pro-3 signal was still invisible. In the early stage of apoptosis, the phosphatidylserine in the inner part of the cell membrane turns over to the surface of the cell membrane, which can be stained by annexin V. To-Pro-3 cannot penetrate live cell membranes but only stains nucleus of dead cells or late-stage apoptotic cells. The result of 5-min laser irradiation indicates that the cells were at the early stage of apoptosis. With continuous irradiation for 10 min, the integrity of the cell membranes was destroyed and the To-Pro-3 signal became obvious. There was almost no luminescence in mitochondria while nucleus luminesce brightly.

It is worth noting that [Ru(phen)_2_dppz]Cl_2_ underwent luminescence translocation from mitochondria to nucleus gradually and the luminescence mainly located in nucleus eventually. These findings suggested that mitochondria-to-nucleus translocation of [Ru(phen)_2_dppz]Cl_2_ during cell apoptosis; furthermore, early and late stages of apoptosis could be distinguished clearly using [Ru(phen)_2_dppz]Cl_2_ as a unique luminescent probe. The ability of [Ru(phen)_2_dppz]Cl_2_ to induce photo-toxicity with real-time imaging could help control the treatment dose that can avoid excessive phototoxicity and minimize potential side effects. If this mitochondria accumulated [Ru(phen)_2_dppz]Cl_2_ were applied to biomedical research in the future, it can be used as a potential and promising theranostic reagent.

In short, *in situ* self-monitoring of apoptosis process was achieved by using the unique ‘photo-triggered nuclear translocation’ property of the Ru complex.

## Discussion

[Ru(phen)_2_dppz]Cl_2_ do not bind unselectively to all forms of nucleic acids. We confirmed that it binds selectively to deoxyribonucleic acid (DNA) rather than ribonucleic acid (RNA) by cellular co-staining experiment and comparing its luminescence with DNA and RNA.

Four Ru complex analogs ([Ru(phen)_3_]Cl_2_, [Ru(bpy)_3_]Cl_2_, [Ru(bpy)_2_dppz]Cl_2_ and [Ru(DIP)_2_dppz]Cl_2_) were tested as mitochondria imaging agents. We found that the three hydrophilic Ru complex analogs but not the more lipophilic [Ru(DIP)_2_dppz]Cl_2_ can stain mitochondria. [Ru(DIP)_2_dppz]Cl_2_ is known to be more lipophilic than the other three Ru complexes, thus we speculated that, the method that targeting of live-cell mtDNA by a Ru(II) complex via dissociation of ion-pair complex with suitable counter-anions is more suitable for hydrophilic Ru(II) complexes than lipophilic Ru(II) complexes.

The chirality of the Ru(II) polypyridyl complex not only influence its cellular DNA luminescent intensity, but also determine its cellular phototoxicity efficiency. The Δ-enantiomer of Ru(II) complex displayed much higher luminescence intensity in nucleus and mitochondria than the Λ-enantiomer. Further we found that the Δ-enantiomer also showed higher cellular phototoxicity efficiency than the Λ-enantiomer.

The mitochondria accumulated [Ru(phen)_2_(dppz)]Cl_2_ can not only induce cell apoptosis by the intrinsic high ROS generation efficiency under visible light irradiation, but also can clearly differentiate healthy and apoptotic cells without any additional agents. Particularly, the luminescent Ru(II) polypyridyl complex can serve as a self-reporter to monitor the cellular photodynamic process, such as where the probe locates, how the phototoxicity performs, and when is the end point of phototoxic effects.

Compared with commercial mitochondrial dyes MTG and PicoGreen, Ru(II) complex showed several advantages (Table 1): it is photo-stable (Supplementary Figure S21) and shows a much larger Stokes shift value with a near infrared red luminescence which is out of the range of cellular autofluorescence, therefore it shows low background. More importantly, different from the mitochondrial membrane staining pattern of MTG, Ru(II) complex stains mitochondrial DNA due to its DNA ‘light-switch’ effect. Besides, Ru(II) polypyridyl complex also stains chromosome DNA in the presence of chlorophenolic counter-anions. It should be noted that PicoGreen stains nuclear DNA and mtDNA simultaneously, but nuclear DNA and mtDNA can be stained separately or simultaneously by Ru(II) complex via formation and dissociation of ion-pairing complex with suitable counter-anions. It is worth noting that Ru(II) complexes, like its close Os(II) analogues, should also be ideal contrast agents for transmission electron microscopy (TEM) imaging, due to their highly electron-dense ruthenium center. Because Ru(II) polypyridyl complex can be imaged by both light microscopy and TEM, we expect that it could be used as a unique cellular mitochondrial DNA probe suitable for correlative light and cryo-electron microscopy (CLEM) studies. Lastly, there is a dramatic difference between the two chiral forms of [Ru(phen)_2_dppz]Cl_2_ for both nuclear and mtDNA imaging, so the chirality of the Ru(II) polypyridyl complex is another very important characteristic as an intracellular DNA structural probe.

**Table 1.** [Ru(phen)_2_dppz]Cl_2_/2,3,4,5-TeCP can be used as live-cell mtDNA probe with many unsurpassed characteristics, as compared with the current commonly used organic mitochondrial probes

	[Ru(phen)_2_dppz]Cl_2_ (2,3,4,5-TeCP)	MitoTracker Green	PicoGreen
Excitation/Emission maximum (nm)	450/620	490/516^a^	502/523^a^
Stokes shift	>150	26	21
Cellular localization	Nuclear DNA and/or mtDNA	Mitochondrial membrane	Nuclear DNA and mtDNA
Photo-bleaching	Low	High	High
TEM	+	–	–
Enantio-selectivity	+	–	–
Binding mode with DNA	Intercalator	–	Intercalator

^a^Molecular probes.

Traditionally, people image mtDNA by expression of the mtDNA-binding protein TFAM (transcription factor A, mitochondrial) or by immunofluorescence technique. However, our Ru complex staining process is much simple and easy-handling as compared to the laborious immunofluorescence technique and protein expression.

Compared with other mitochondrial targeting Ru and Ir complexes reported, the selective targeting of live-cell mitochondrial DNA by [Ru(phen)_2_dppz]Cl_2_ via dissociation of ion-pairing complex with suitable counter-anions in this work showed several new findings (Table 2). Most of previous work studied anticancer ability of Ru or Ir complexes, but not clear about their exact locations in mitochondria: mtDNA, intermembrane space or protein (50–55). The dinuclear Ru complex was found to be located in the intermembrane space of mitochondria, not the central lumen where mtDNA is located (50). No direct evidence was provided that Ru-MPP can bind mtDNA in cells, they just co-localized Ru-MPP with MitoTracker and imaged by fluorescence microscopy (FM) and luminescence lifetime imaging microscopy (LLIM) (53). In this work, [Ru(phen)_2_dppz]Cl_2_ was found to bind specifically with mitochondrial DNA but not intermembrane space not only by biochemical methods such as agarose gel electrophoresis, mitochondrial membrane stained with Tomo20-dronpa, competitive binding with mtDNA intercalator PicoGreen, but also by super-resolution optical microscopy techniques such as SIM and STED in live cells, which even can realize counting the copy number of mtDNA in one mitochondrion.

**Table 2.** [Ru(phen)_2_dppz]Cl_2_/2,3,4,5-TeCP can be used as live-cell mtDNA probe with many unsurpassed characteristics, compared with other Ru or Ir complexes

	Location in mitochondria	SRM imaging	Evidence of binding with mtDNA	Excitation/Emission maximum (nm)
[Ru(phen)_2_dppz]^2+^ (through dissociation of ion-pair complex)	mtDNA	SIM, STED	SRM, PicoGreen, gel electrophoresis, etc.	488/620
[(phen)_2_Ru(tpphz)Ru(phen)_2_]^4+a^	intermembrane space	SIM, STED	–	488/630 680
Ru-MPP^b^	mtDNA	–	FM, LLIM	457/628
[Ru(dppz)_2_(CppH)]^2+c^	–	–	–	458/620
Cyclometalated Ir(III) complexes^d^	mtDNA	–	PicoGreen	385/590

^a^Ref. (50). ^b^Ref. (53). ^c^Ref. (51). ^d^Ref. (52).

## Conclusions

In conclusion, through formation and dissociation of ion-pairing complex with chlorophenolic counter-anions, [Ru(phen)_2_dppz]Cl_2_ can be used as a unique probe to image both nucleus and mitochondria by super-resolution optical microscopy. Especially, [Ru(phen)_2_dppz]Cl_2_ was found to bind specifically with mitochondrial DNA in live cells by SIM and STED. We further found that the luminescence intensity in the mitochondria and photo-sensitizing effects of the Δ-enantiomer of [Ru(phen)_2_dppz]Cl_2_ is higher than that of Λ-enantiomer. In situ self-monitoring of photodynamic effects of the ruthenium complex was achieved by the translocation of the ruthenium complex from mitochondria to nucleus during cell apoptosis. This is the first report on enantio-selective targeting, super-resolution imaging of live-cell mitochondrial DNA and cellular photo-sensitizing by a Ru(II) complex via formation and dissociation of ion-pair complex with suitable counter-anions.

## Supplementary Material

gkad799_Supplemental_Files

## Data Availability

The data underlying this article are available in the article and in its online supplementary material.
