# Supplementary material for: Unprecedented enantio-selective live-cell mitochondrial DNA super-resolution imaging and photo-sensitizing by the chiral ruthenium polypyridyl DNA “light-switch”
Source: Nucleic Acids Res. 2023 Nov 2;51(22):11981–98. doi: 10.1093/nar/gkad799 (PMC10711558; doi:10.1093/nar/gkad799)
Supplement: gkad799_Supplemental_Files [file gkad799_supplemental_files.zip › NARMito SI clean.pdf]

Supporting Information

Unprecedented Enantio-Selective Live-Cell Mitochondrial DNA Super-Resolution Imaging and Photo-Sensitizing by the Chiral Ruthenium Polypyridyl DNA "Light-Switch"

Rong Huang[1,2], Chun-Hua Huang[2]*, Jing Chen[2], Zhu-Ying Yan[2], Miao Tang[2], Jie Shao[2], Kaiyong Cai[1], Ben-Zhan Zhu[2,3]*

[1] Key Laboratory of Biorheological Science and Technology, Ministry of Education, College of Bioengineering, Chongqing University, Chongqing, 400044, China.
[2] State Key Laboratory of Environmental Chemistry and Ecotoxicology, Research Center for Eco-Environmental Sciences, and University of Chinese Academy of Sciences, Chinese Academy of Sciences, Beijing, 100085, China.
[3] Linus Pauling Institute, Oregon State University, Corvallis, OR 97331, USA.
*To whom correspondence should be addressed. Tel: 86-10-62849030; Fax: 86-10-62923563; Email: chhuang@rcees.ac.cn; bzhu@rcees.ac.cn.

RESULTS AND DISCUSSION

Video S1. Z slice images of mitochondria in a Hela cell stained by the Ru complex and MTG.

Video S2. Volume view of mitochondria in a Hela cell stained by the Ru complex.

Video S3. Real-time dynamic change a cell stained by the mitochondrial accumulated Ru complex.

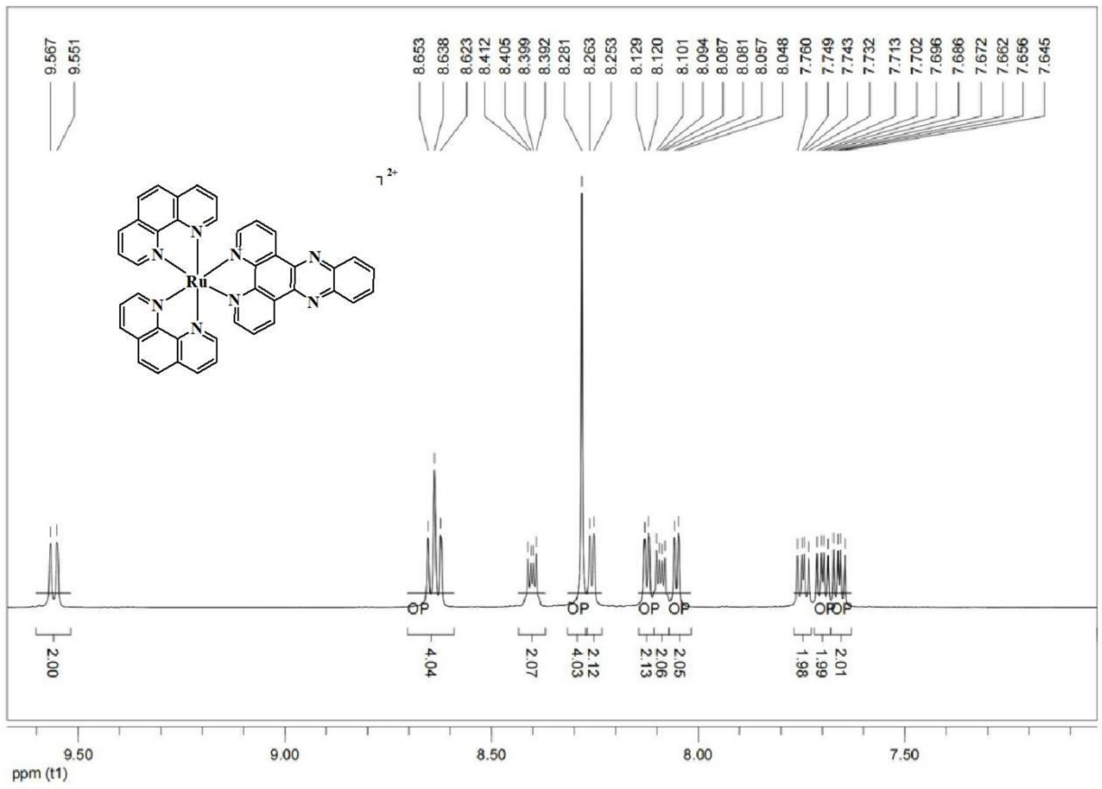

Figure S1. ^{1}H NMR spectrum of $[Ru(phen)_2dppz]^{2+}$. NMR (Bruker, Germany) was performed to get the structure of $[Ru(phen)_2dppz]^{2+}$. ^{1}H NMR (400 MHz, acetonitrile-d_3) δ 9.57(s, 2H), δ 8.65 (s, 4H), δ 8.41 (s, 2H), δ 8.28 (s, 4H), δ 8.26 (s, 2H),δ 8.13 (s, 2H),δ 8.10 (s, 2H),δ 8.06 (s, 2H),δ 7.76 (s, 2H), 7.71 (s, 2H), 7.67 (s, 2H).

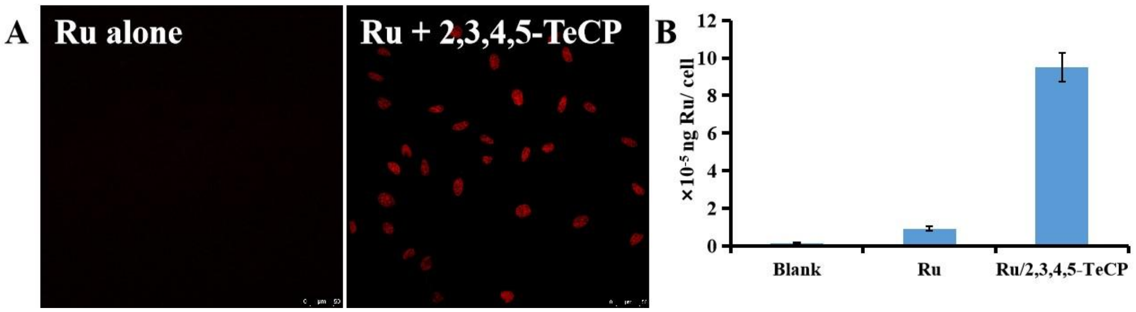

Figure S2. 2,3,4,5-TeCP facilitated the cellular uptake of [Ru(phen)$_2$dppz]Cl$_2$. Cells incubated with 0.1 mM [Ru(phen)$_2$dppz]Cl$_2$ with or without 2,3,4,5-TeCP for 0.5 h, then observed by CLSM (A) or measured by ICP-MS (B). Scale bar: 10 μm.

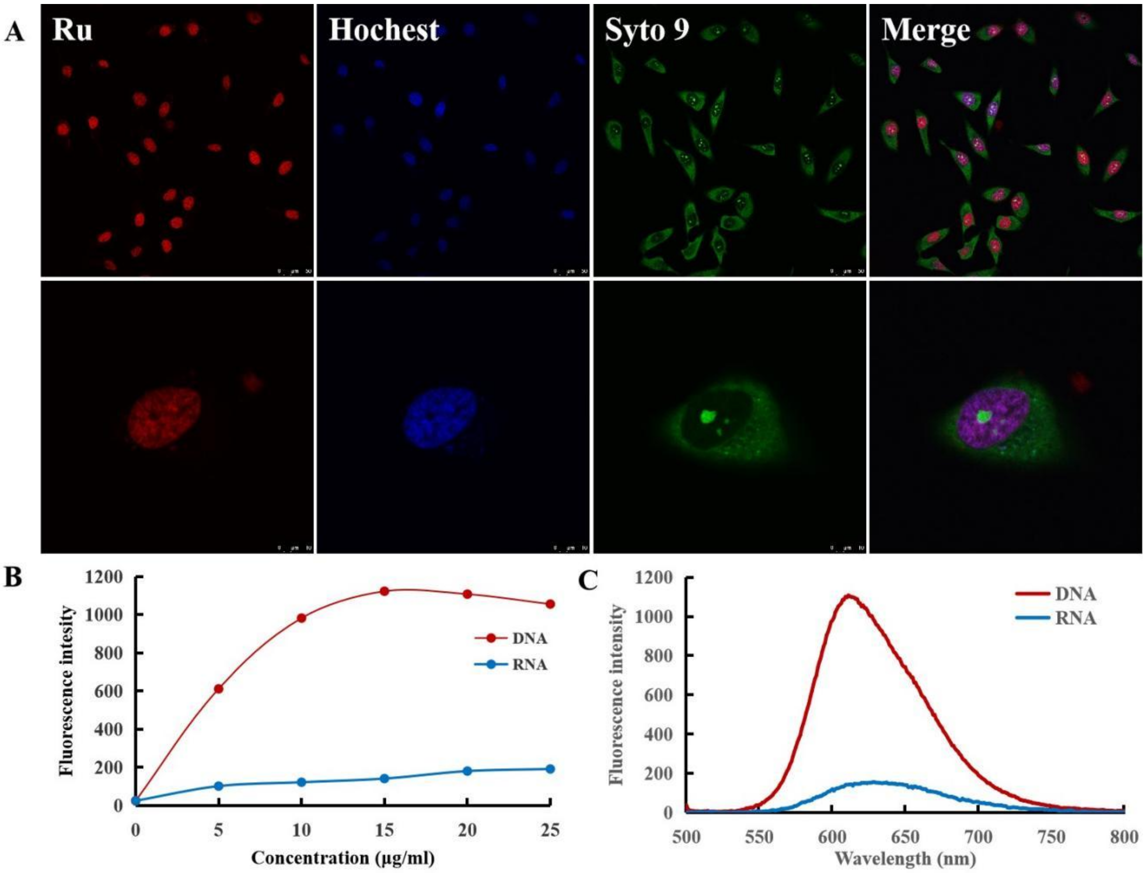

Figure S3. [Ru(phen)$_2$dppz]Cl$_2$ was found to bind selectively to DNA rather than RNA. A, Co-staining of [Ru(phen)$_2$dppz]Cl$_2$ (red, left) with DNA specifific dye Hochest (blue, centre), general nucleic acid dye SYTO 9 (green, centre) and the overlay images (right). Scale bar: 50 μm (up) and 10 μm (down). B, Luminescence intensity (in 611 nm) of 10 μM Ru (10 mM Tris-HCl, pH 7.4) in the presence of yeast RNA up to 25 μg/ml compared to their luminescence intensity when saturated with ctDNA. Data represent mean ± S.D. of three independent experiments. C, Representative luminescence intensity curve of [Ru(phen)$_2$dppz]Cl$_2$ with 15 μg/ml DNA and RNA.

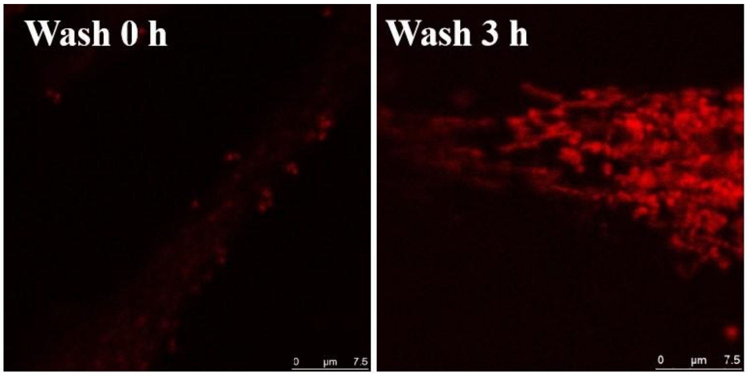

Figure S4. The emission of [Ru(phen)$_2$dppz]Cl$_2$ in cytoplasm after washed for 3 h is much brighter than that washed for 0 h. Scale bar: 7.5 μm.

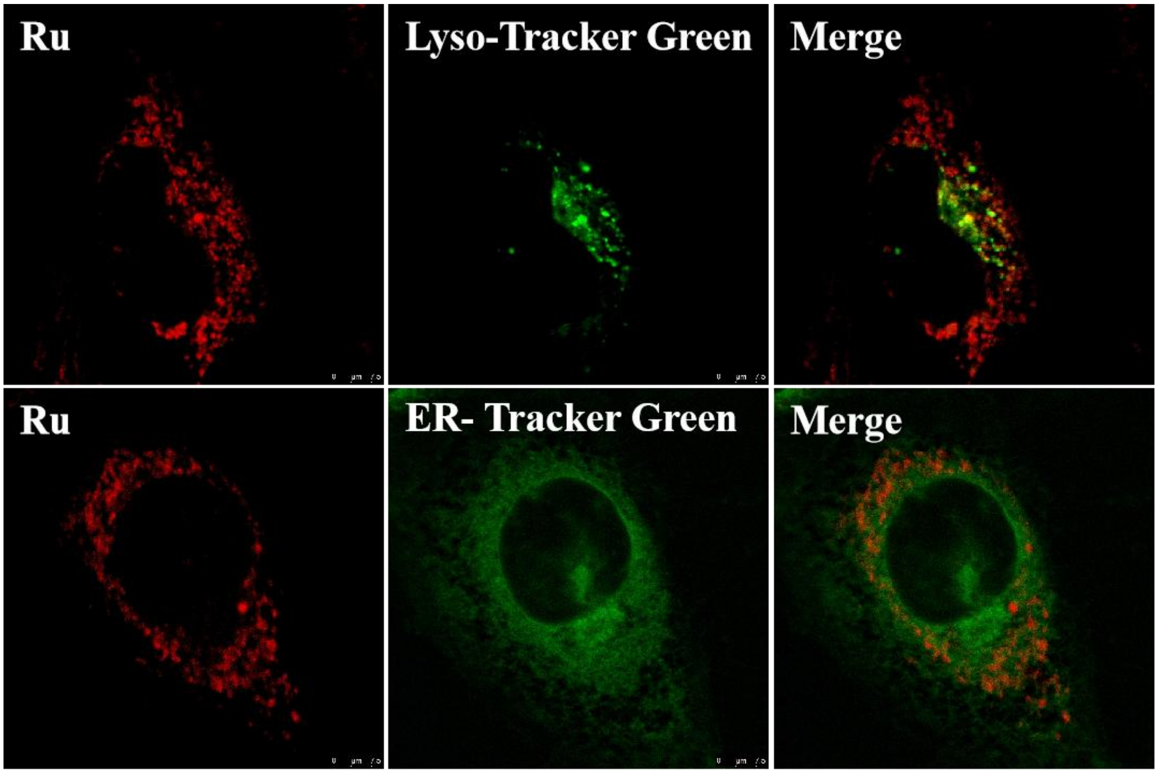

Figure S5. Co-staining of [Ru(phen)$_2$dppz]Cl$_2$ with lysosome and endoplasmic reticulum stain: Lyso-Tracker Green and ER-Tracker Green. Note the low co-localization of [Ru(phen)$_2$dppz]Cl$_2$ with the green Lyso-Tracker and ER-Tracker emission from the cytoplasm of cells. Scale bar: 7.5 μm.

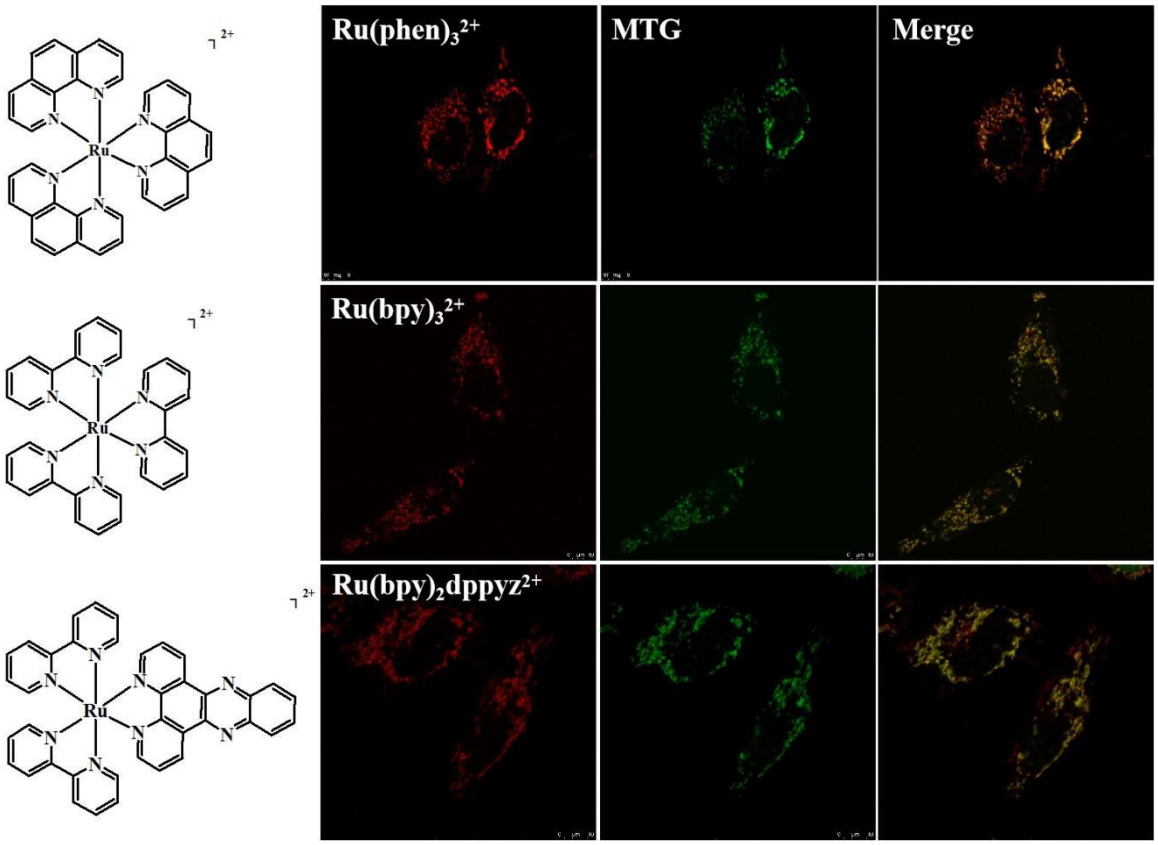

Figure S6. Chemical structure of Ru complexes and representative CLSM images of cells stained with Mito-Tracker Green (MTG) (green) and co-stained with [Ru(phen)$_2$dppz]Cl$_2$ analogs (red). The luminescence was found to be mainly inside the mitochondria when Hela cells were incubated with 0.2 mM [Ru(phen)$_3$]Cl$_2$/[Ru(bpy)$_3$]Cl$_2$ (FBS free medium) and 0.4 mM 2,3,4,5-TeCP for 30 min, or 0.1 mM [Ru(bpy)$_2$dppz]Cl$_2$ and 0.3 mM 2,3,4,5-TeCP for 1 h, and then incubated with fresh medium for 3 h. Scale bar: 50 μm.

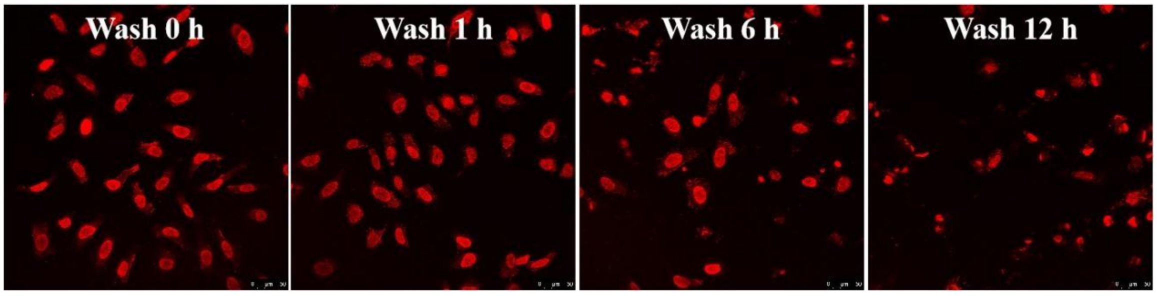

Figure S7. [Ru(DIP)$_2$dppz]Cl$_2$ can not relocate from nuclei to mitochondria after washing. Hela cells were incubated with 30 μM [Ru(DIP)$_2$dppz]Cl$_2$ and 100 μM 3,5-DCP for 1 h, and then incubated with fresh medium for 1 h, 3 h and 12 h. Scale bar: 50 μm.

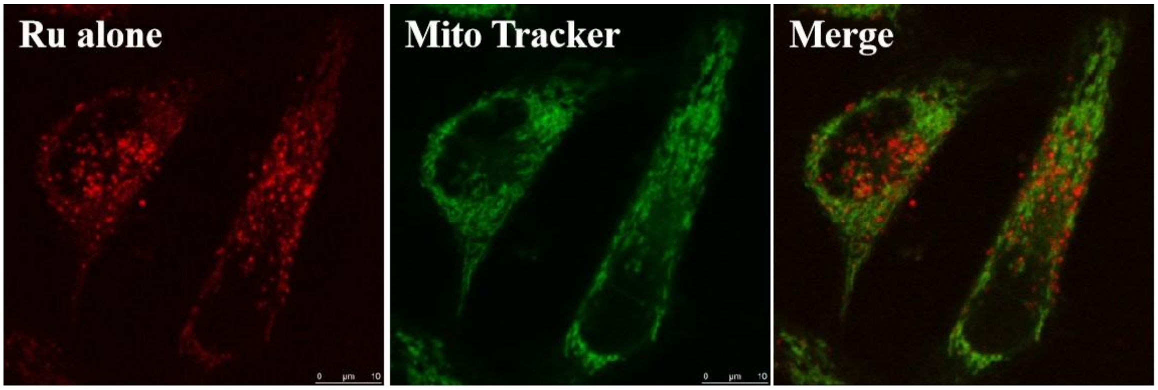

Figure S8. Cells incubated with high concentration [Ru(phen)$_2$dppz]Cl$_2$ alone showed Ru distribution throughout cytoplasm, not concentrated in mitochondria. Cells incubated with 0.3 mM Ru for 2 h. Scale bar: 10 μm.

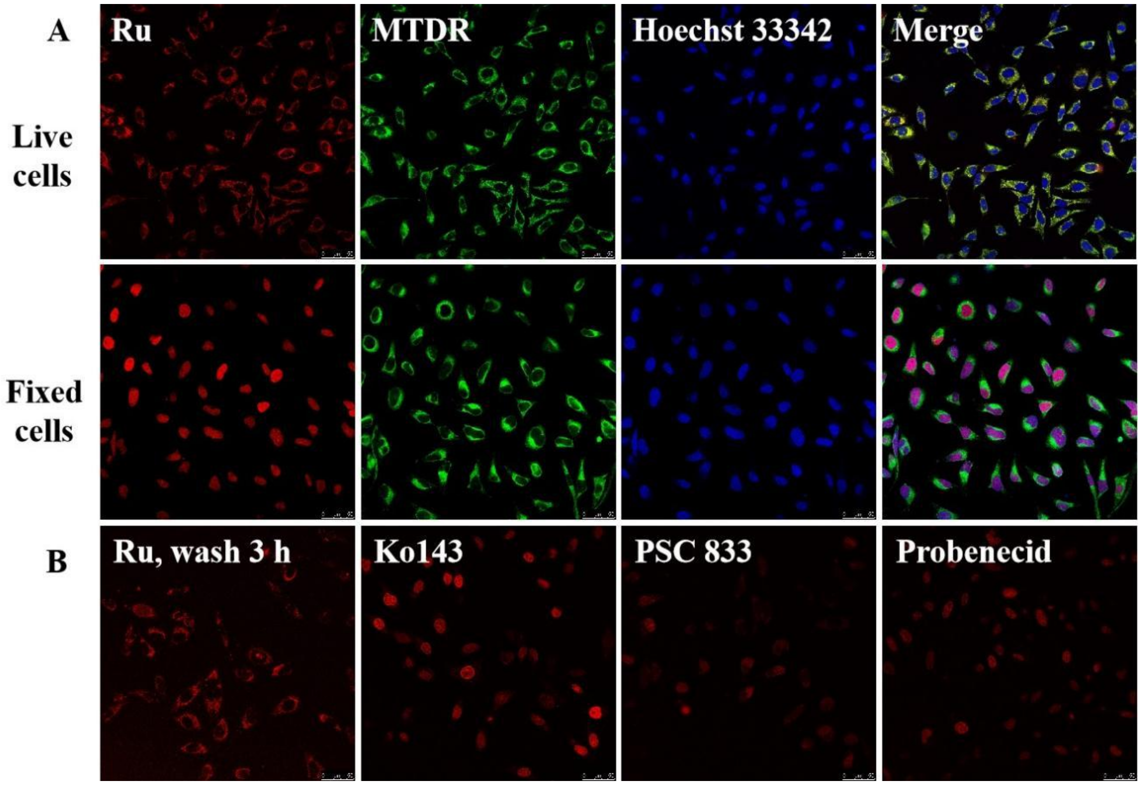

Figure S9. [Ru(phen)$_2$(dppz)]Cl$_2$ effluxed out of cell nucleus by the active ATP dependent ABC transporter proteins. (A) Live cells and fixed cells incubated with [Ru(phen)$_2$(dppz)]Cl$_2$ and 2,3,4,5-TeCP for 0.5 h, washed and refilled with fresh medium for 3 h, then stained with 0.2 μM MTDR and 10 μg/ml Hoechest33342 for 20 min. For fixed cells, cells fixed with 4% paraformaldehyde at room temperature for 20 min. (B) ABC transport protein inhibitors prevented the efflux of [Ru(phen)$_2$dppz]Cl$_2$ from nuclei to mitochondria. Cells were incubated with [Ru(phen)$_2$(dppz)]Cl$_2$ and 2,3,4,5-TeCP for 0.5 h, washed and refilled with fresh medium with/ without 100 mM Ko143, 4 mM

PSC-833 or 10 mM probenecid for 3 h, images were captured by confocal microscopy. Scale bar: 25 μm.

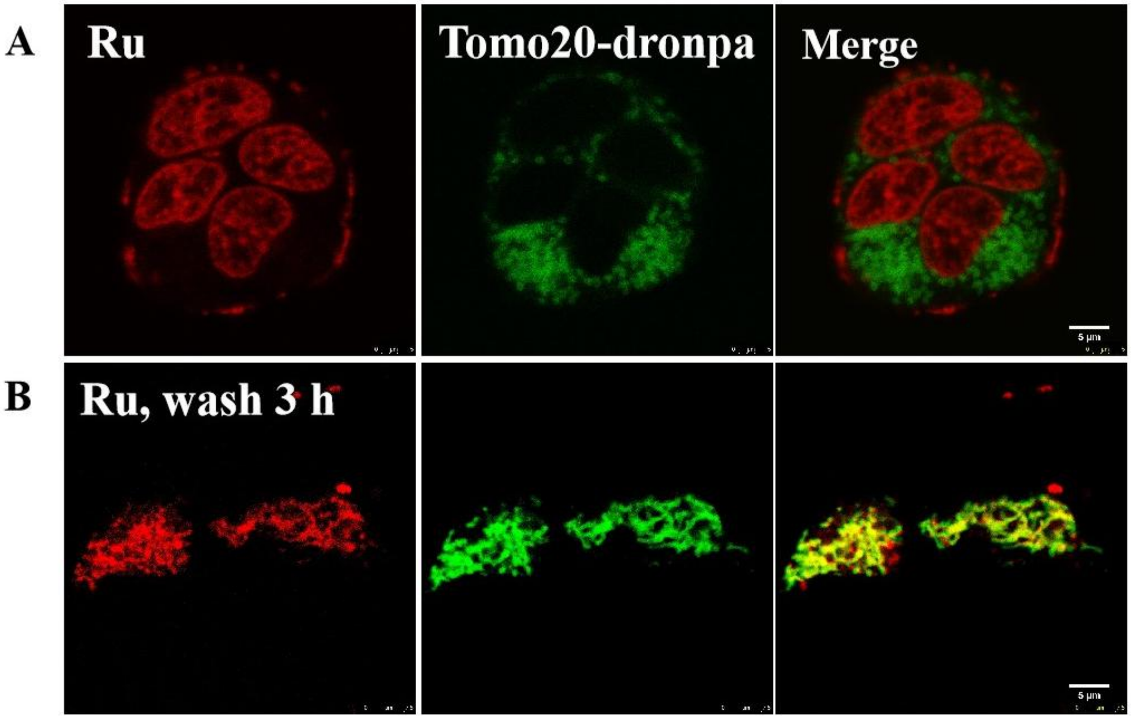

Figure S10. Co-staining [Ru(phen)$_2$dppz]Cl$_2$ (Red) with mitochondrial outer membrane protein Tomo20-dronpa (green). A: Representative CLSM images of Hela cells stained with Ru and co-stained with Tomo20-dronpa at two different treatments: Cells stained with Ru and 2,3,4,5-TeCP for 0.5 h showed nuclear localization, which is different from the mitochondrial staining of Tomo20-dronpa. B: Cells stained with Ru and 2,3,4,5-TeCP for 0.5 h and then incubated with fresh medium showed mitochondrial staining, and co-localization maps displaying the co-localized objects in yellow. Scale bar: 5 μm.

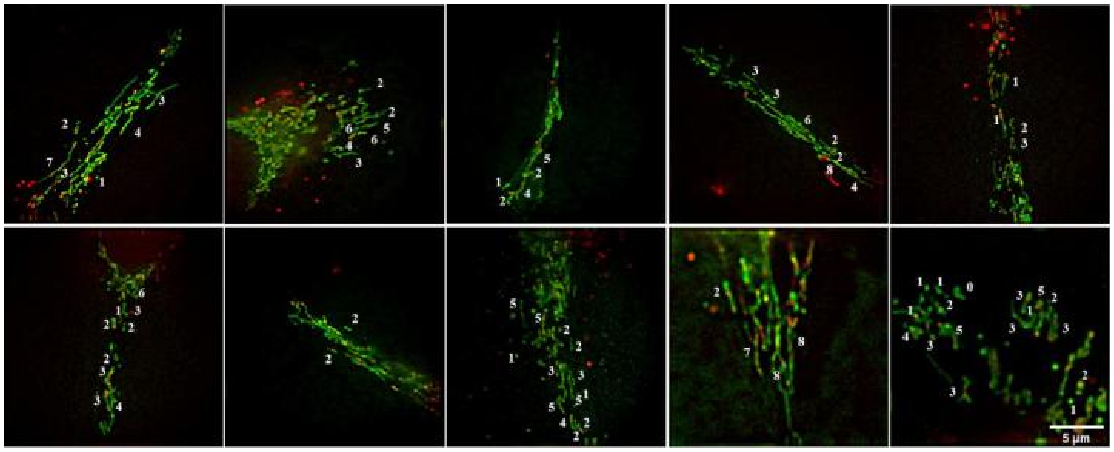

Figure S11. Deconvolution images of [Ru(phen)$_2$dppz]Cl$_2$ co-stained with mitochondrial outer membrane protein Tomo20-dronpa for mtDNA counting. Mitochondrial DNA-positive structures were counted manually on images captured by 3D high-resolution microscopy of conventional mode. The total number of nucleoids was thus determined after the merge of 5 focal planes to a single image. Red spots indicated the mtDNA stained by [Ru(phen)$_2$dppz]Cl$_2$ and green strips represented the mitochondrial membrane stained by Tomo20-dronpa. Scale bar: 5 μm.

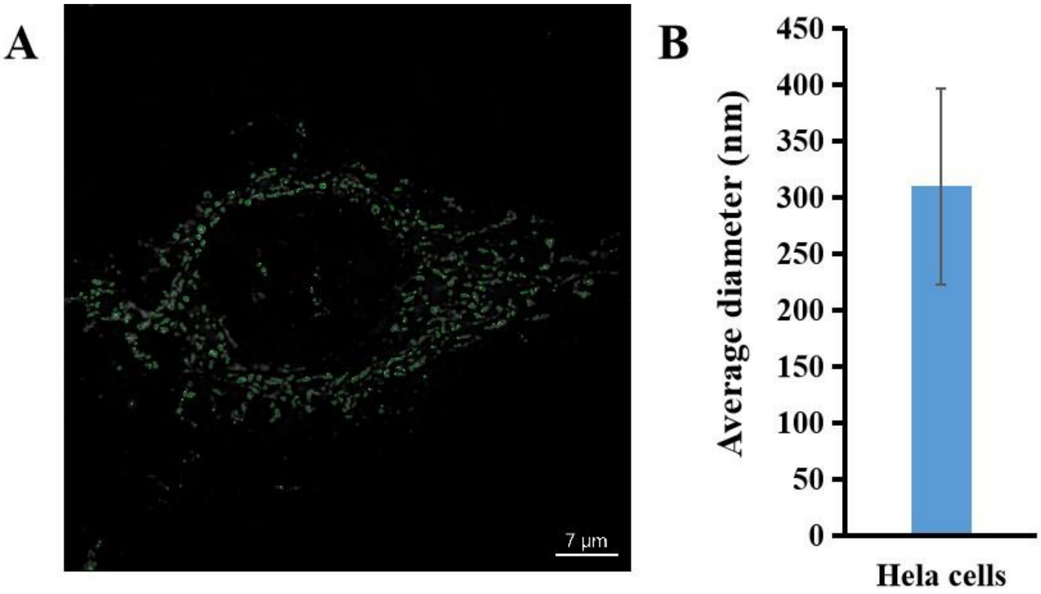

Figure S12. Measuring mitochondrial nucleoids mean size in live Hela cells by mitochondria accumulated Ru complex. (A) Representative cell labeled with [Ru(phen)$_2$dppz]Cl$_2$ used for for mtDNA size calculation. The background grey image is the emission from mtDNA staining [Ru(phen)$_2$dppz]Cl$_2$. The green surfaces denote mtDNA for size calculation by. Scale bar: 7 μm. (B) Sizes of live Hela cell nucleoids labeled with [Ru(phen)$_2$dppz]Cl$_2$ determined by multi-SIM imaging and "number of voxels" analysis of IMARIS software.

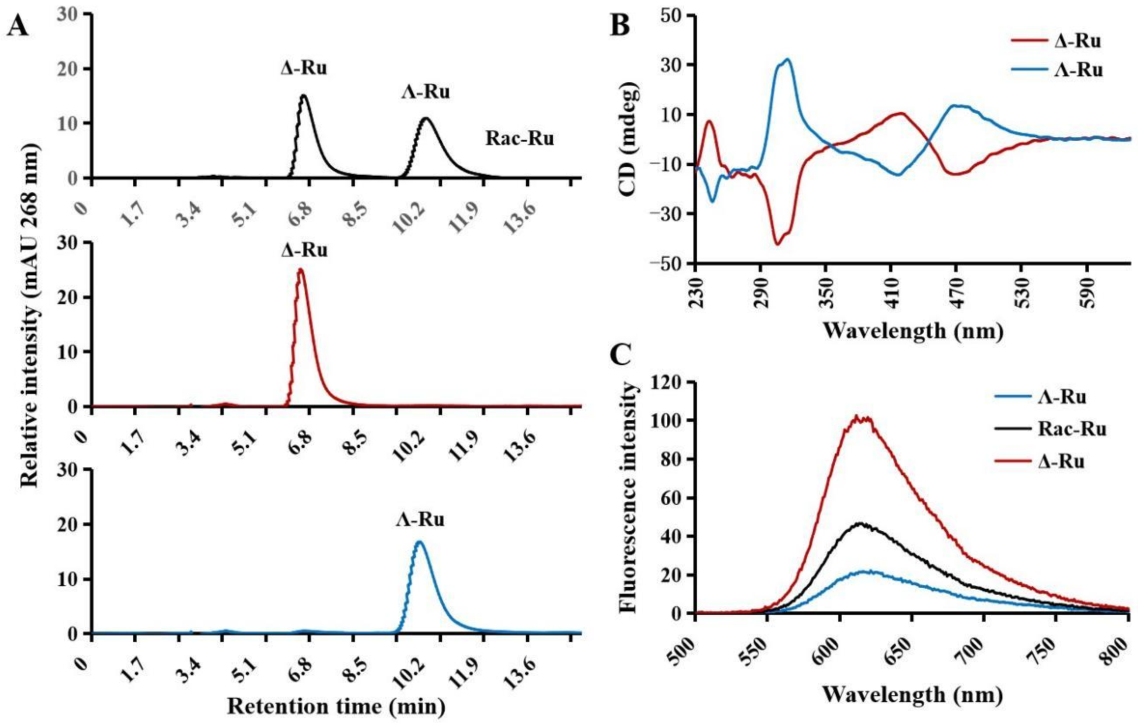

Figure S13. Two pure enantiomers of [Ru(phen)$_2$(dppz)]Cl$_2$ were separated successfully. (a) The seperation of the two enantiomers of [Ru(phen)$_2$(dppz)]Cl$_2$ by a CYCLOBOND I 2000 DMP high-performance liquid chromatography (HPLC) column (250 × 21.2 mm, 5 µm, Sigma) on a Agilent 1260 HPLC. (b) Assignment of the two fractions (50 µM) was further confirmed by circular dichroism. (c) Δ-enantiomer (50 µM) showed higher emission when binding with ctDNA (200 µM) than that of Λ-Ru (50 µM).

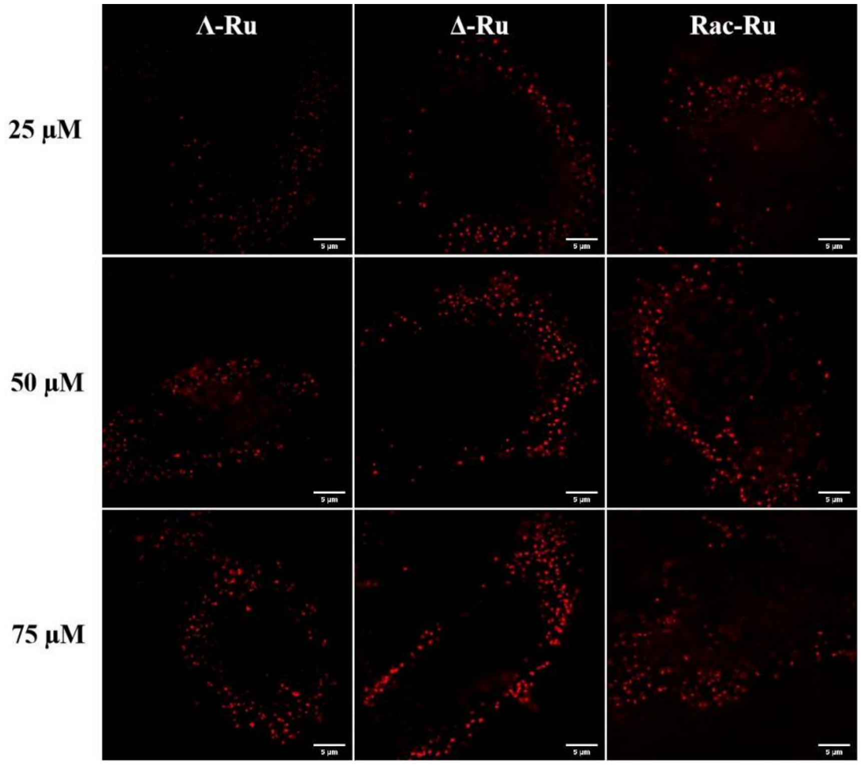

Figure S14. SIM images of Hela cells stained with different concentrations of racemic Ru(II) complexes and its two chiral forms. Cells stained with different concentrations (25 µM, 50 µM or 75 µM) of Ru and 2,3,4,5-TeCP for 0.5 h and then incubated with fresh medium for 3 h. Scale bar: 5 µm.

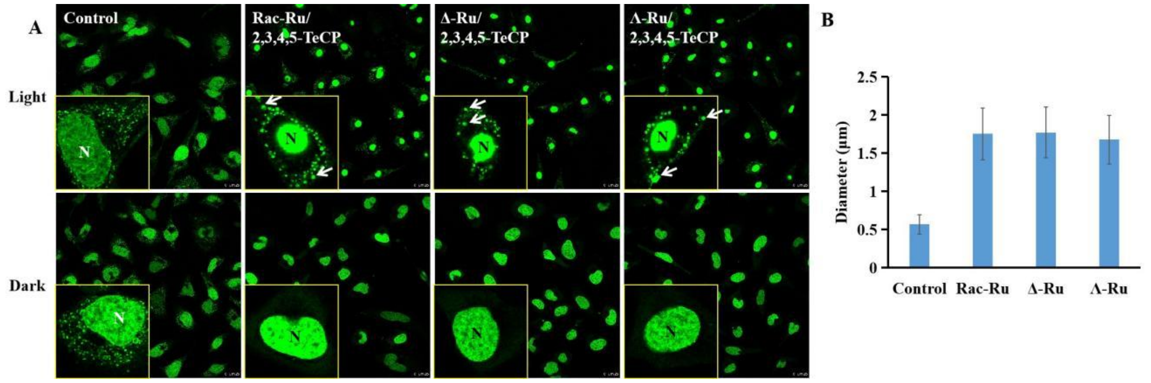

Figure S15. Observation of nucleoid remodeling/mtDNA damage from irradiation of mitochondrial accumulated [Ru(phen)$_2$(dppz)]Cl$_2$ with PicoGreen staining. (A) Hela cells treated with [Ru(phen)$_2$(dppz)]Cl$_2$/2,3,4,5-TeCP for 0.5 h, washed with PBS and incubated with fresh medium for 3 h, then irradiated with or without a 450 nm light (30 mW/cm^2, 20 min). After another 24 hours incubation, cells stained with PicoGreen and imaged by CLSM. The yellow border images at the bottom left show a single cell enlarged from each images. Left N: nucleus, white arrows: representative remodeled nucleoids. Scale bar: 25 µm. (B) 10 nucleoids were randomly selected from four images

of cells after irradiation in A for diameter measurement.

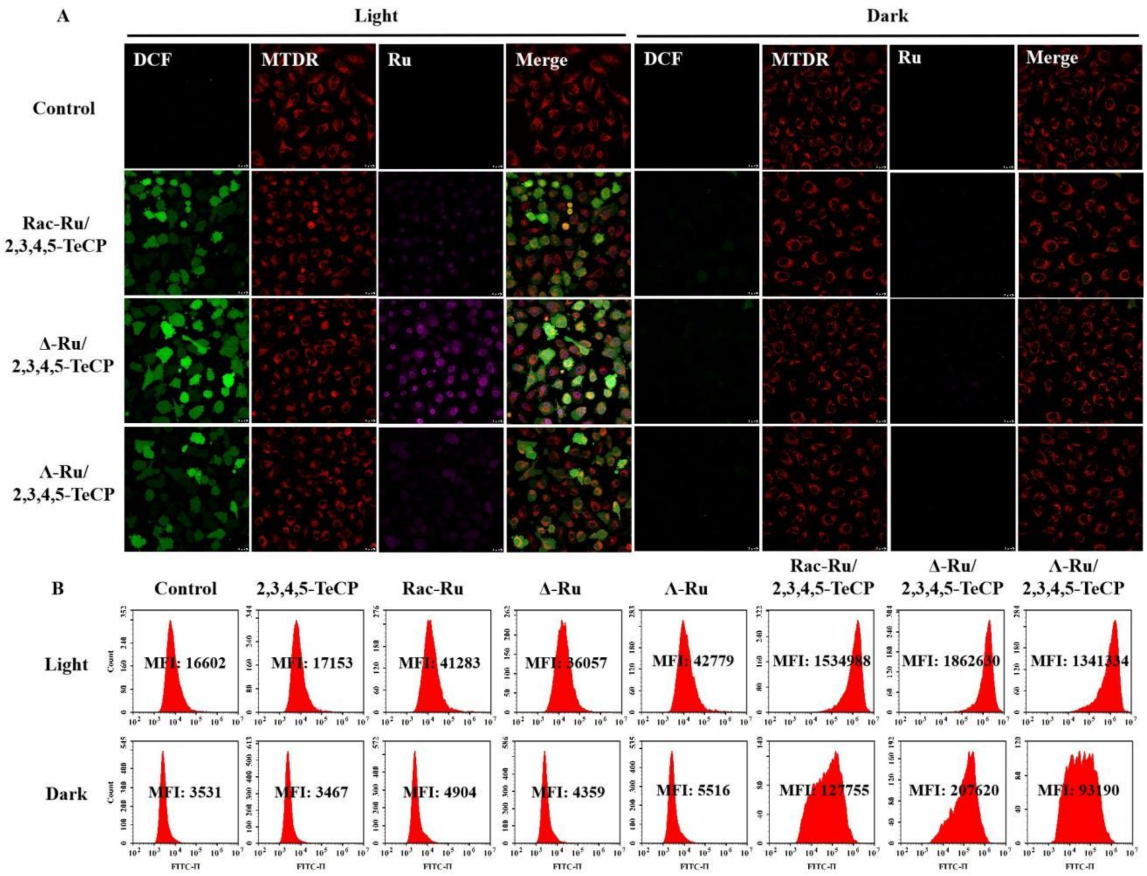

Figure S16. Quantification intracellular ROS levels in cells treated with Ru complex. (A) CLSM images of mitochondrial ROS production caused by Ru treatment. Scale bar: 25 μm. (B) Flow cytometric analysis of ROS generation caused by Ru-mediated PDT. Hela cells were incubated with $[Ru(phen)_2(dppz)]Cl_2$/2,3,4,5-TeCP for 0.5 h, washed with PBS and incubated with fresh medium for 3 h, then irradiated with a 450 nm light (30 mW/cm2, 20 min). The cells were stained with H_2DCFDA and MTDR and analyzed by confocal microscopy, or stained with H_2DCFDA and analyzed by flow cytometry.

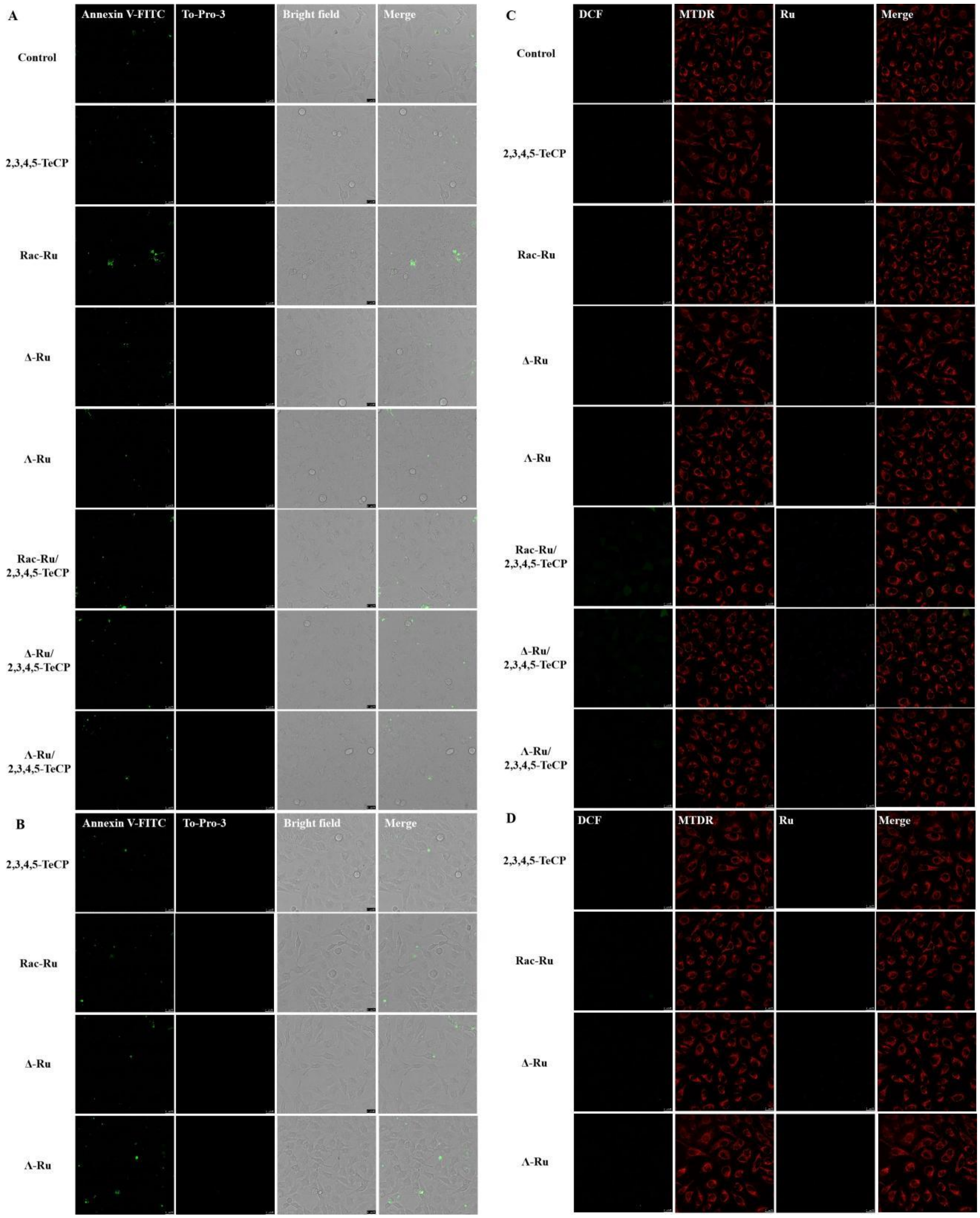

Figure S17. Annexin V-FITC/To-Pro-3 staining and ROS detection CLSM images of negative control cells. (A and B) CLSM images of cells stained with Annexin-V-FITC and To-Pro-3. (C and D) CLSM images of mitochondrial ROS production. Hela cells were incubated with [Ru(phen)$_2$(dppz)]Cl$_2$/2,3,4,5-TeCP for 0.5 h, washed with PBS and incubated with fresh medium for 3 h, then irradiated with (B, D) or without (A, C) a 450 nm light (30 mW/cm^2, 20 min). The cells were stained with H$_2$DCFDA and MTDR and

analyzed by CLSM. Or the cells incubated for another 24 h, then stained with Annexin V-FITC/To-Pro-3 and analyzed by CLSM. Scale bar: 25 μm.

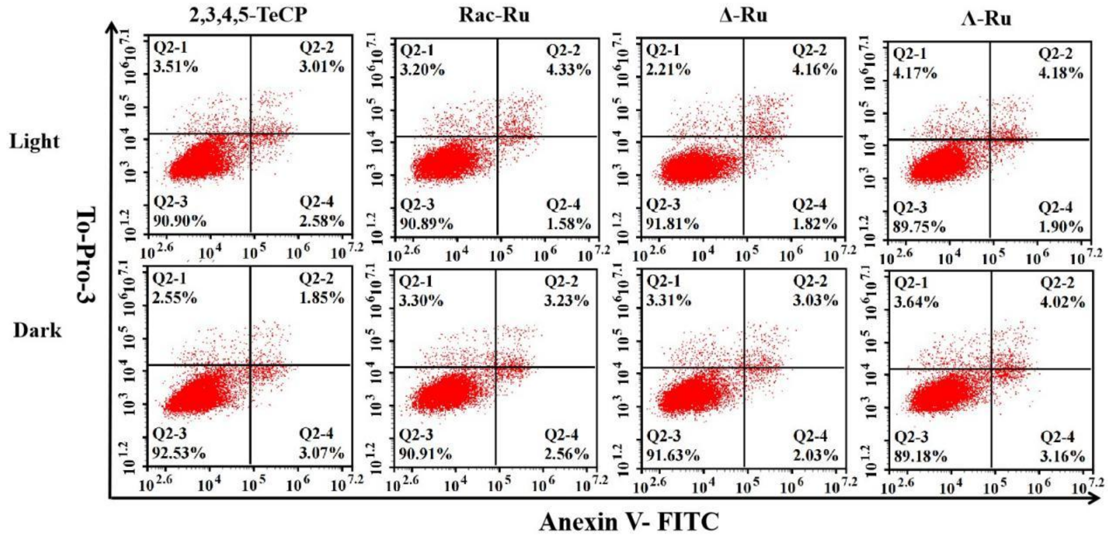

Figure S18. FACS detection of apoptosis in cells pretreated with [Ru(phen)$_2$(dppz)]Cl$_2$ or 2,3,4,5-TeCP alone. After treatment with Ru (100 μM) or 2,3,4,5-TeCP (300 μM) for 0.5 h, cells were rinsed with PBS for 3 times, refilled with new fresh medium and incubated for 3 h, then cells were exposed to light irradiation (450 nm, 30 mW/cm^2, 20 min). Cells were stained with Annexin V-FITC (10 μM) and To-Pro-3 (0.5 μM) for 15 min after incubation for another 24 h, then detected by FACS.

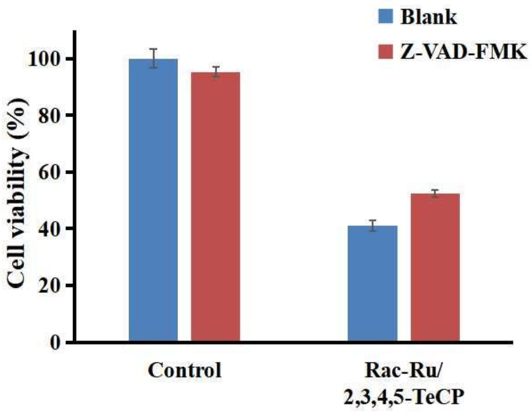

Figure S19. Theeffects of Z-VAD-FMK on the cytotoxicity of Ru in Hela cells upon irradiation. After treatment with Ru (100 μM)/2,3,4,5-TeCP (300 μM) for 0.5 h, cells were rinsed with PBS for 3 times, refilled with new fresh medium and incubated for 3 h, then cells were exposed to light irradiation (450 nm, 30 mW/cm^2, 30 min). Z-VAD-FMK (50 μM) added 1 h before irradiation were used for caspase inhibition.

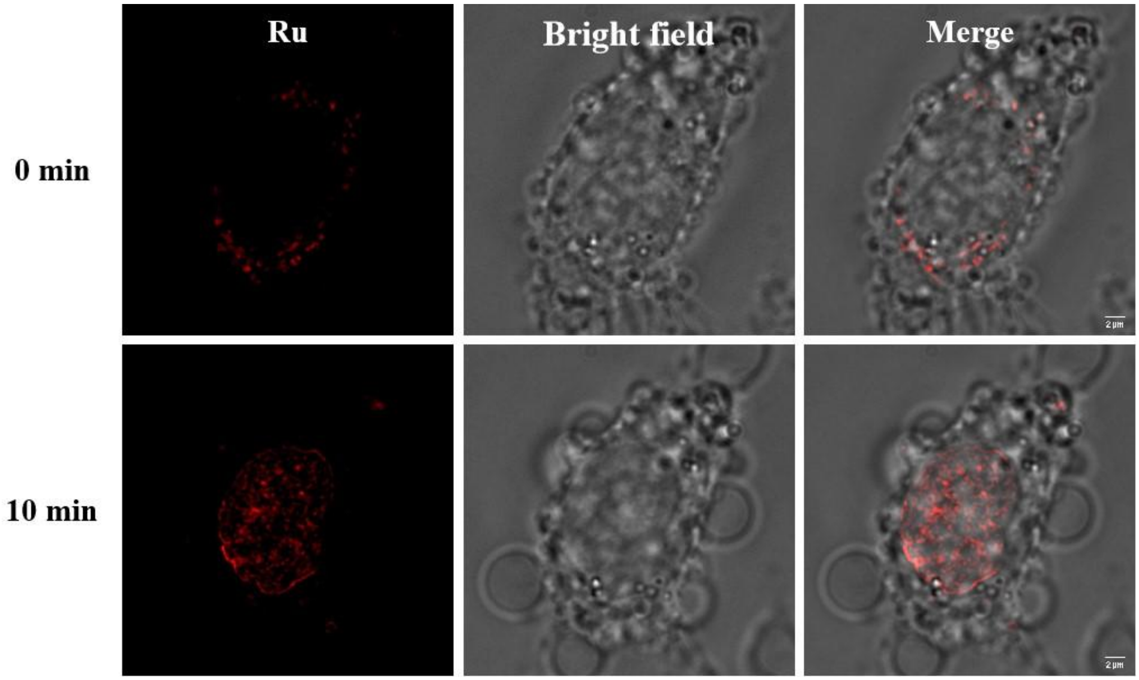

Figure S20. Multi-SIM imaging of Cells before and after light irradiation for 10 min stained with [Ru(phen)$_2$dppz]Cl$_2$/2,3,4,5-TeCP.

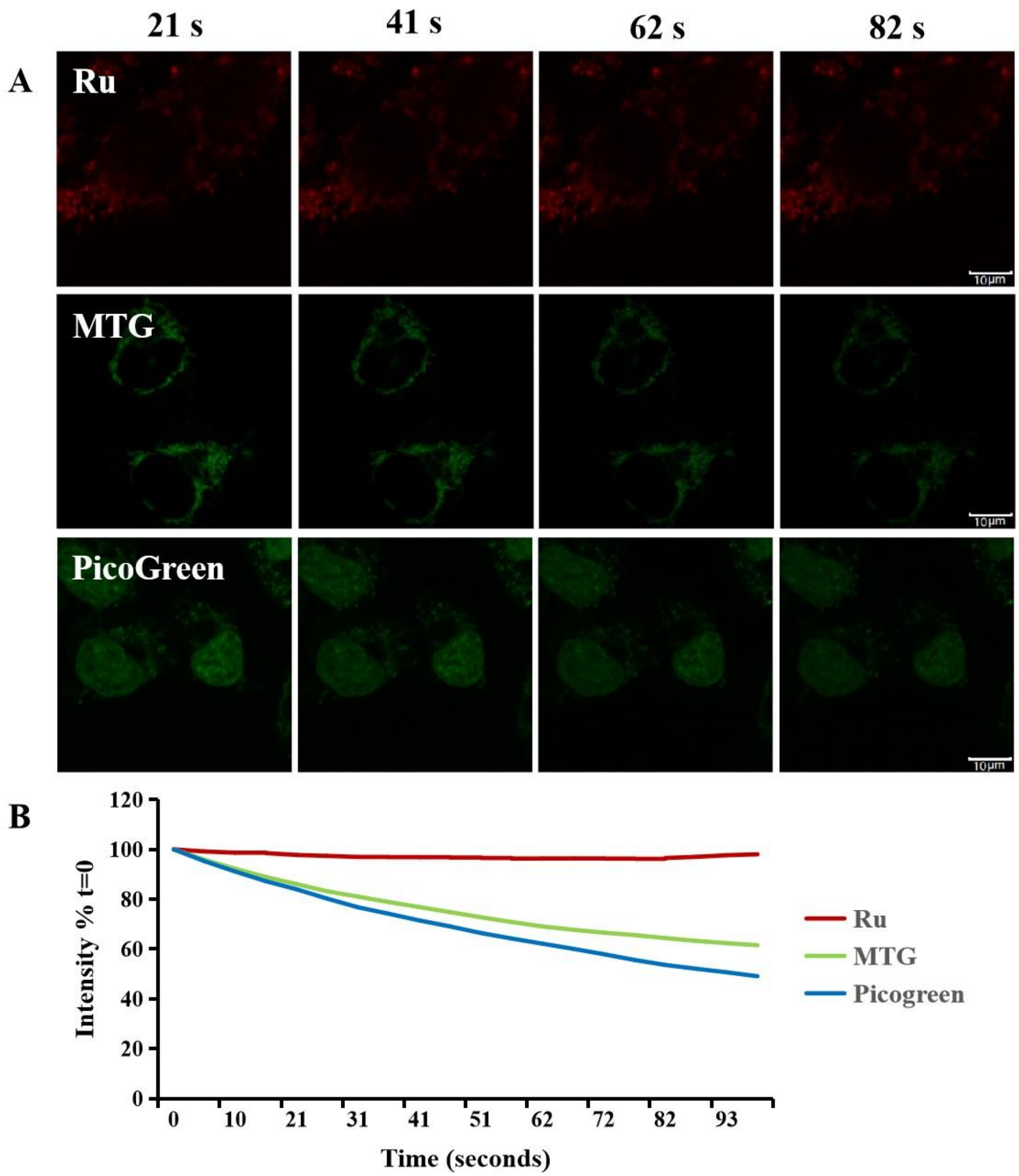

Figure S21. Photo-stability of [Ru(phen)$_2$dppz]Cl$_2$ as compared with MTG and PicoGreen in cells. (A) Time series showing luminescence of [Ru(phen)$_2$dppz]Cl$_2$ (100 µM) incubated with 2,3,4,5-TeCP (300 µM) in cellular mitochondria under constant laser exposure with no observable photo-bleaching over 98 seconds exposure time (top, red). In contrast to MTG (20 nM), which demonstrates faster fading than [Ru(phen)$_2$dppz]Cl$_2$ (middle, green). The same experiment was conducted with PicoGreen (bottom, green). All excited with 488 nm. (B) Relative in cellulo luminescence intensity profile time series of [Ru(phen)$_2$dppz]Cl$_2$ (red) demonstrating stronger photo-stability over MTG (green) and PicoGreen (blue). Scale bar: 10 µm.
